# Supplementary material for: Longitudinal development of category representations in ventral temporal cortex predicts word and face recognition
Source: Nat Commun. 2023 Dec 4;14:8010. doi: 10.1038/s41467-023-43146-w (PMC10696026; doi:10.1038/s41467-023-43146-w)
Supplement: Supplementary file 1 — Supplementary Information [file 41467_2023_43146_MOESM1_ESM.pdf]

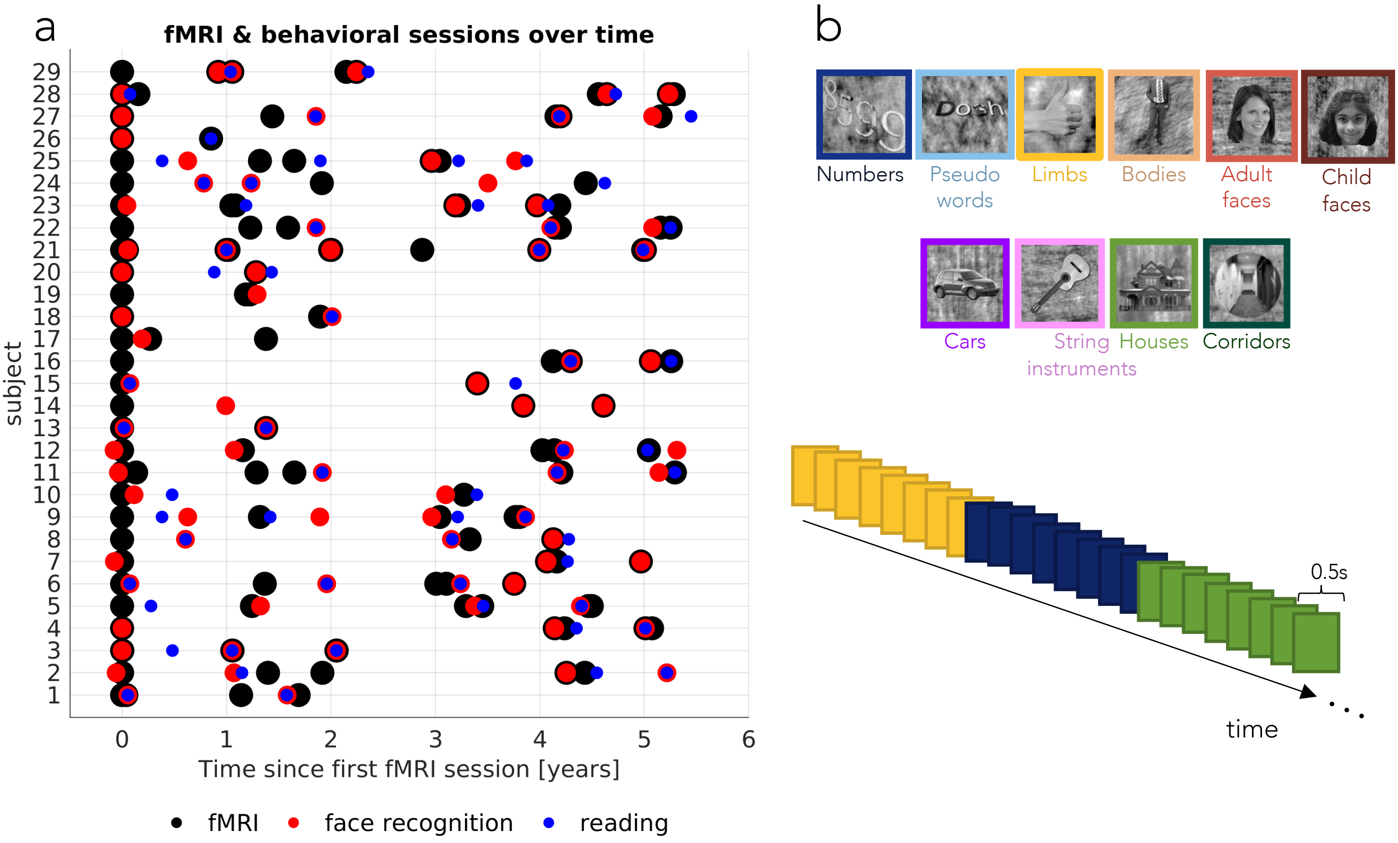

Fig S1. Data acquisition and experimental design

(A) fMRI and behavioral sessions in each participant over time. The scatter plot illustrates the number and temporal sequence of fMRI and behavioral sessions in each child included in the analyses. Each row is a participant. Sessions are plotted relative to each child's first fMRI session. (B) Example stimuli and fMRI experimental design. *Top:* example stimuli for each of the 10 categories, which can be grouped into five domains (characters, body parts, faces, objects, and places). Stimuli of faces were replaced to comply with copyright but resemble those shown in the experiment. *Bottom:* Schematic illustrating the fMRI paradigm. Stimuli were shown at a rate of 2Hz and were presented in random order 4s blocks. Participants were instructed to fixate on a central dot and indicate with a button press when an image that only contained a scrambled background appeared.

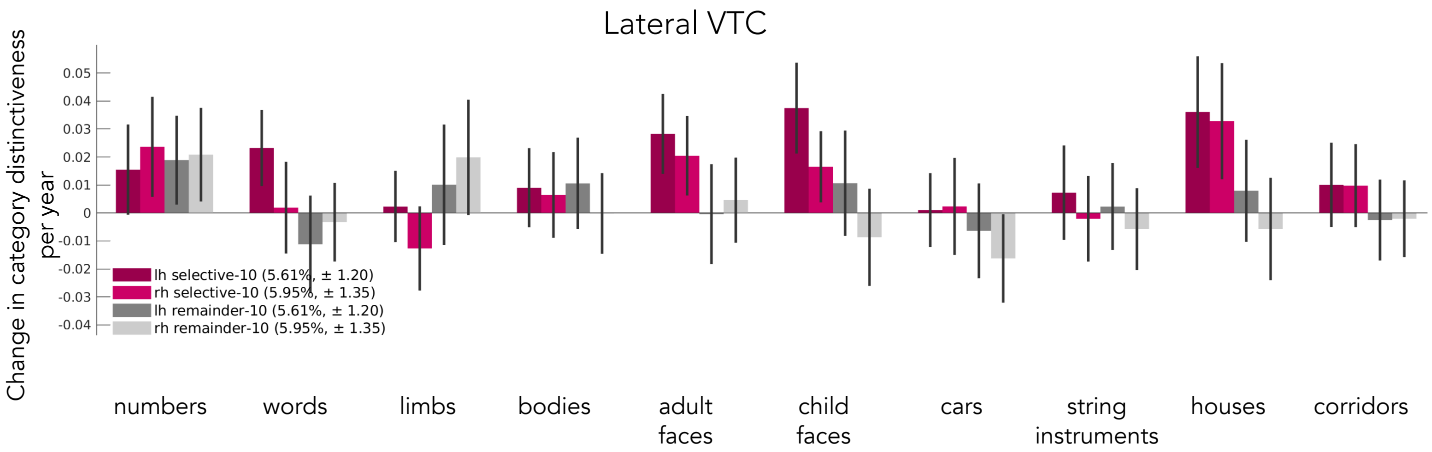

Fig S2. Development of distinctiveness in subsets of the union of selective voxels and the non-selective voxels in lateral VTC that are matched for variance explained.

Bars indicate the change in category distinctiveness per year (LMM relating distinctiveness to age and tSNR, with participant as a random effect, n=128 sessions, 29 children) in different subsets of voxels of lateral VTC that are matched for variance explained. *Maroon bars:* a subset of the size of 300 voxels out of the union of voxels in lateral VTC defined in each session. *Gray bars:* a subset of 300 voxels out of the non-selective voxels of lateral VTC defined in each session.

Category-selectivity was computed by contrasting responses to a category vs. all other categories except the other category from the same domain (e.g., numbers vs. all other categories except words). A voxel was defined as selective to a category when t>3. *Darker colors*: left hemisphere. *Lighter colors*: right hemisphere. *Error bars:* 95% CI. If the CI does not cross the y=0 line, the change in distinctiveness is significantly different than 0. Detailed statistics for this figure can be found in the data repository (see Methods, Data availability).

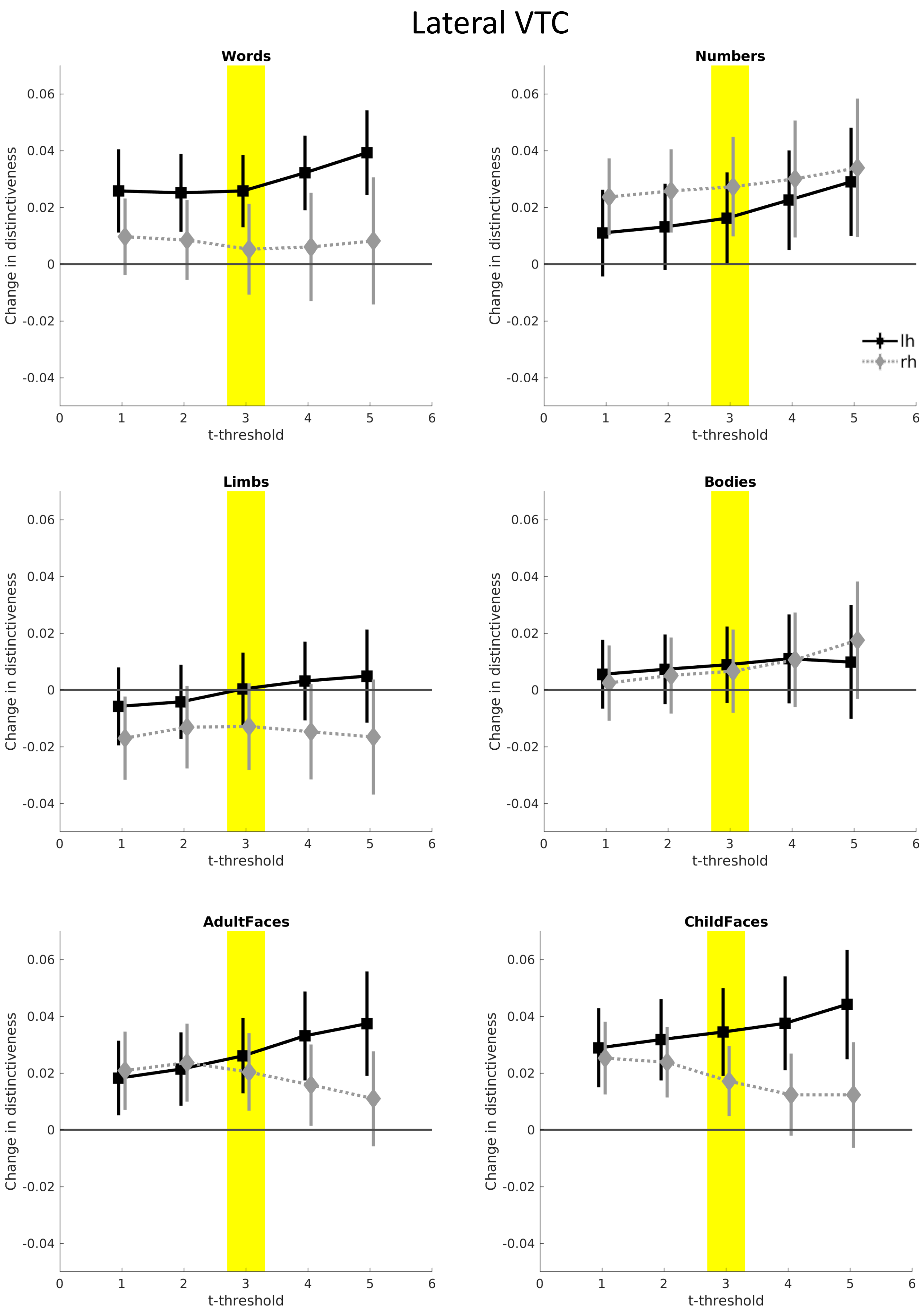
Lateral VTC
Words
Numbers
Limbs
Bodies
AdultFaces
ChildFaces
Change in distinctiveness
t-threshold
lh
rh
0.06
0.04
0.02
0
-0.02
-0.04
0
1
2
3
4
5
6

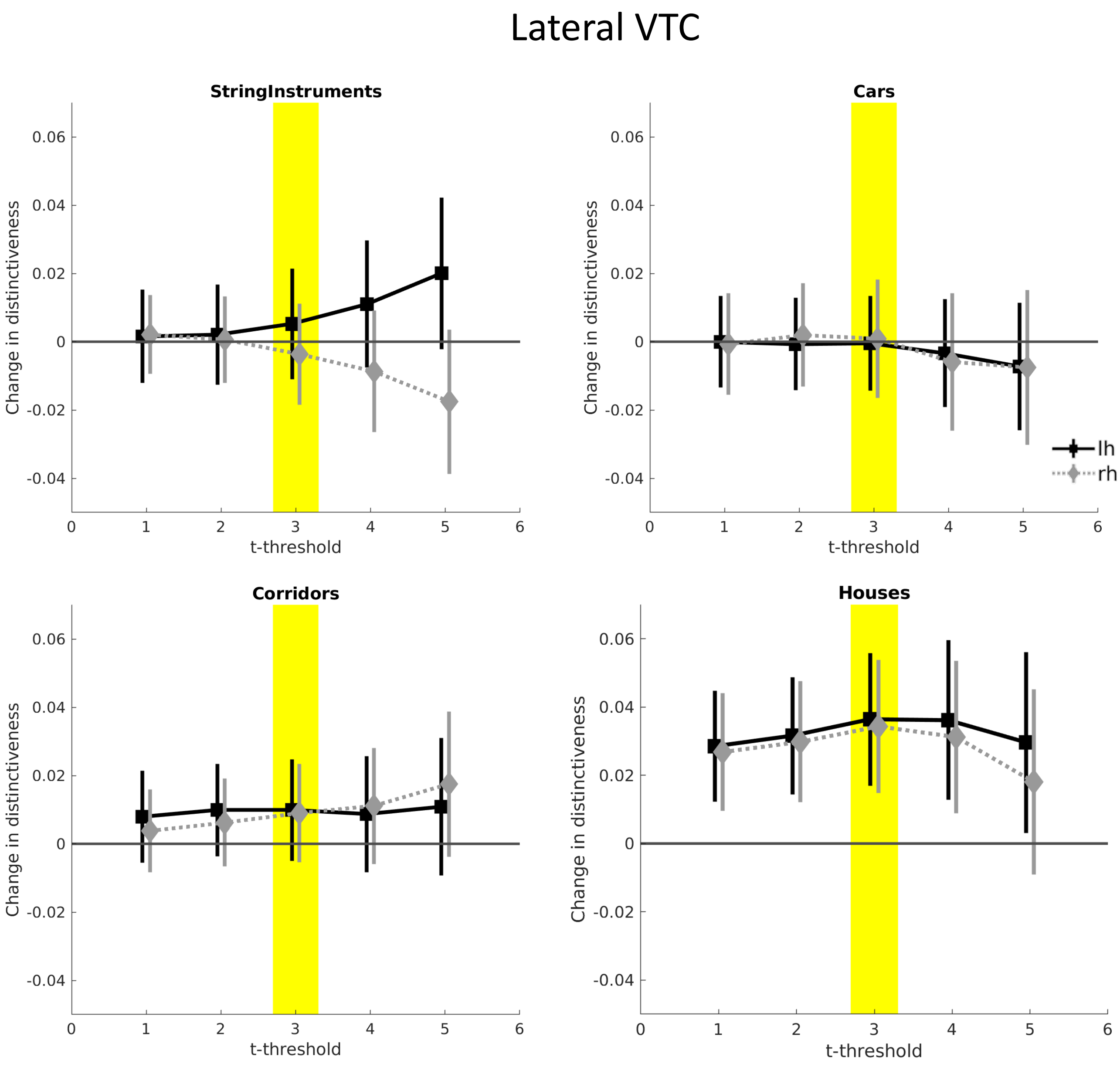

Fig S3. Changes in distinctiveness in the union of selective voxels in lateral VTC are largely stable across different t-values to define category-selective voxels.
Black squares (lh) and gray diamonds (rh) indicate the change in category distinctiveness per year (LMM relating distinctiveness to age and tSNR, with participant as a random effect, n=128 sessions, 29 children) in the union of selective voxels in lateral VTC. The x-axis shows the t-value that was used as a threshold to define a voxel as selective. The union of selective voxels was defined by first defining category-selective voxels to all categories and then taking the union of all these voxels. For each category, selectivity was computed by contrasting responses to a category vs. all other categories except the other category from the same domain (e.g., numbers vs. all other categories except words). The results shown for a t-value ≥ 3 (highlighted in yellow) correspond to the main analysis presented in Fig. 2 of the manuscript. Detailed statistics for this figure can be found in the data repository (see Methods, Data availability).

Medial VTC

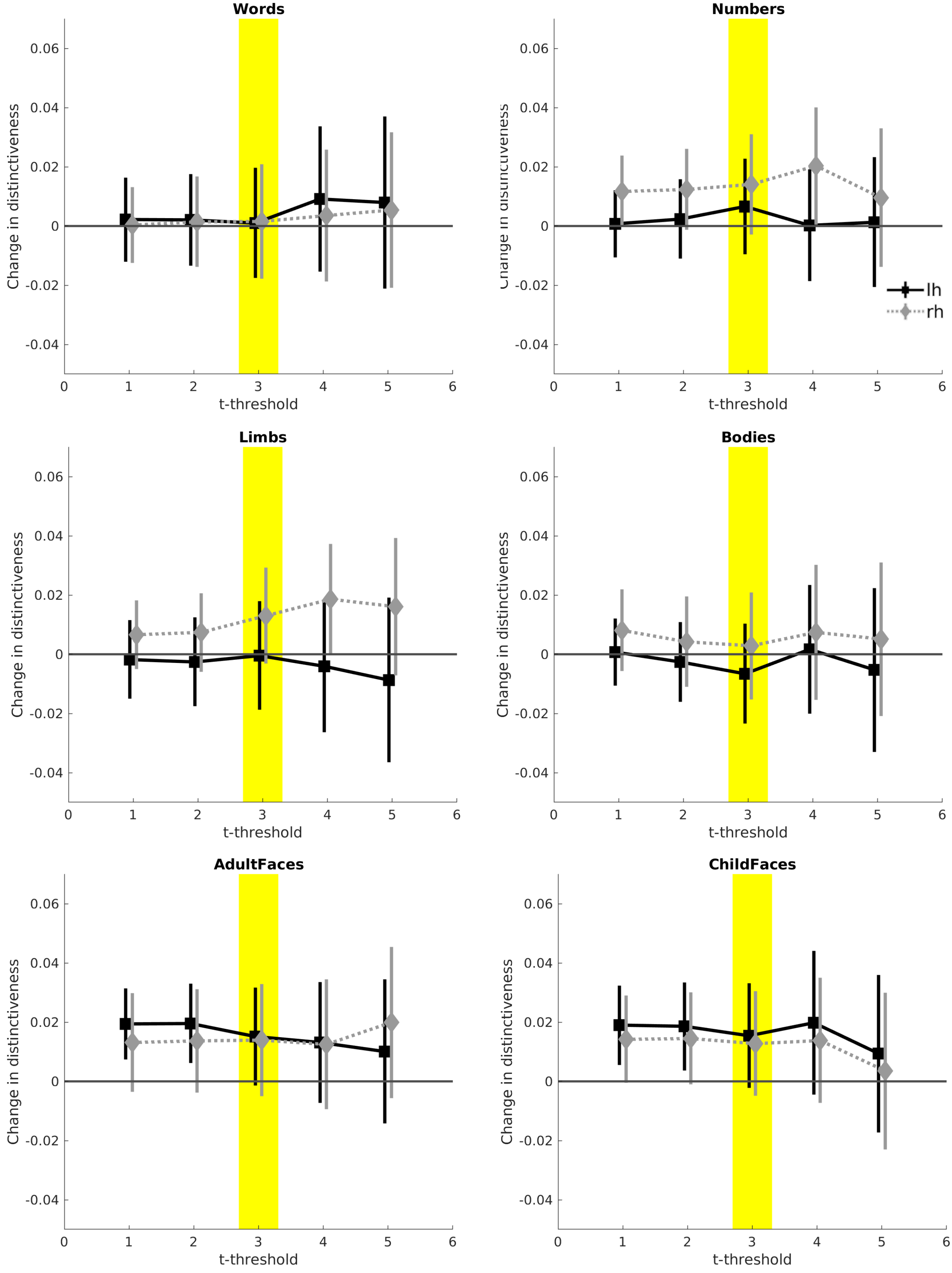
Words
Numbers
Limbs
Bodies
AdultFaces
ChildFaces
Change in distinctiveness
t-threshold
lh
rh
0.06
0.04
0.02
0
-0.02
-0.04
0
1
2
3
4
5
6

Medial VTC

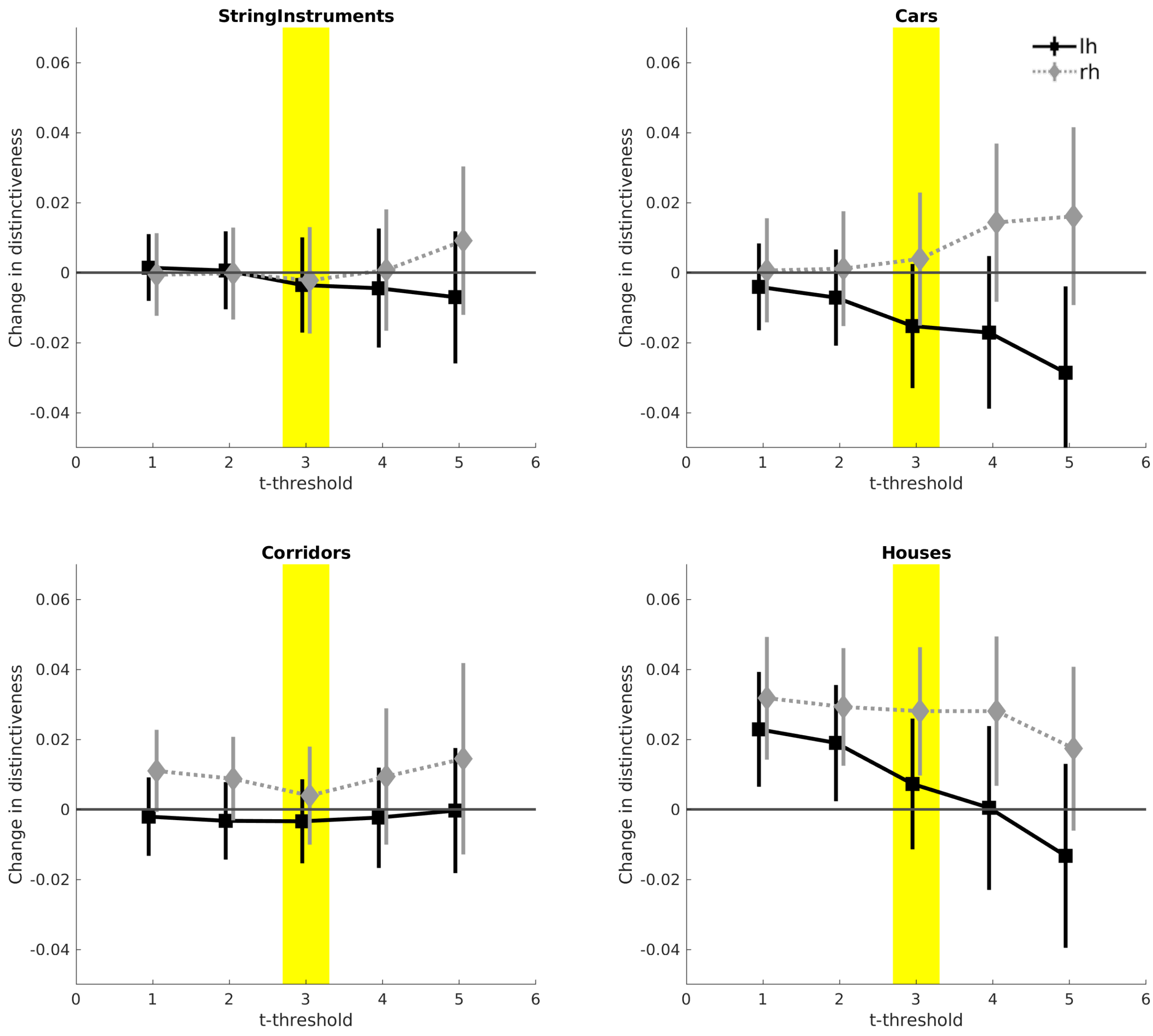

Fig S4. Changes in distinctiveness in the union of selective voxels in medial VTC are largely stable across different t-values to define category-selective voxels.

Black squares (lh) and gray diamonds (rh) indicate the change in category distinctiveness per year (LMM relating distinctiveness to age and tSNR, with participant as a random effect, n=128 sessions, 29 children) in the union of selective voxels in medial VTC. The x-axis shows the t-value that was used as a threshold to define a voxel as selective. The union of selective voxels was defined by first defining category-selective voxels to all categories and then taking the union of all these voxels. For each category, selectivity was computed by contrasting responses to a category vs. all other categories except the other category from the same domain (e.g., numbers vs. all other categories except words). The results shown for a t-value t-value ≥ 3 (highlighted in yellow) correspond to the main analysis presented in Fig. 2 of the manuscript. Detailed statistics for this figure can be found in the data repository (see Methods, Data availability).

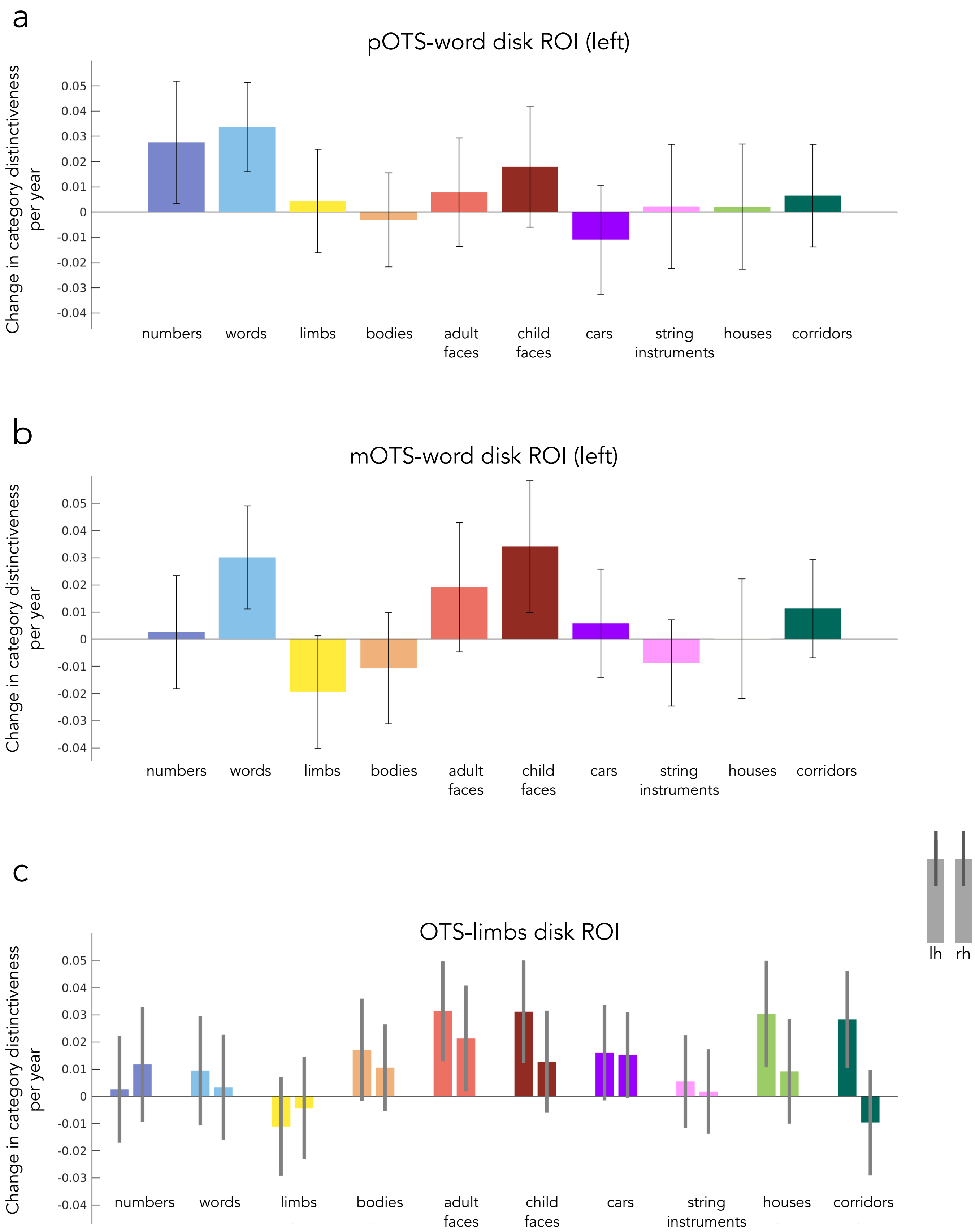
a
pOTS-word disk ROI (left)
Change in category distinctiveness per year
0.05
0.04
0.03
0.02
0.01
0
-0.01
-0.02
-0.03
-0.04
numbers
words
limbs
bodies
adult faces
child faces
cars
string instruments
houses
corridors
b
mOTS-word disk ROI (left)
Change in category distinctiveness per year
0.05
0.04
0.03
0.02
0.01
0
-0.01
-0.02
-0.03
-0.04
numbers
words
limbs
bodies
adult faces
child faces
cars
string instruments
houses
corridors
c
OTS-limbs disk ROI
lh
rh
Change in category distinctiveness per year
0.05
0.04
0.03
0.02
0.01
0
-0.01
-0.02
-0.03
-0.04
numbers
words
limbs
bodies
adult faces
child faces
cars
string instruments
houses
corridors

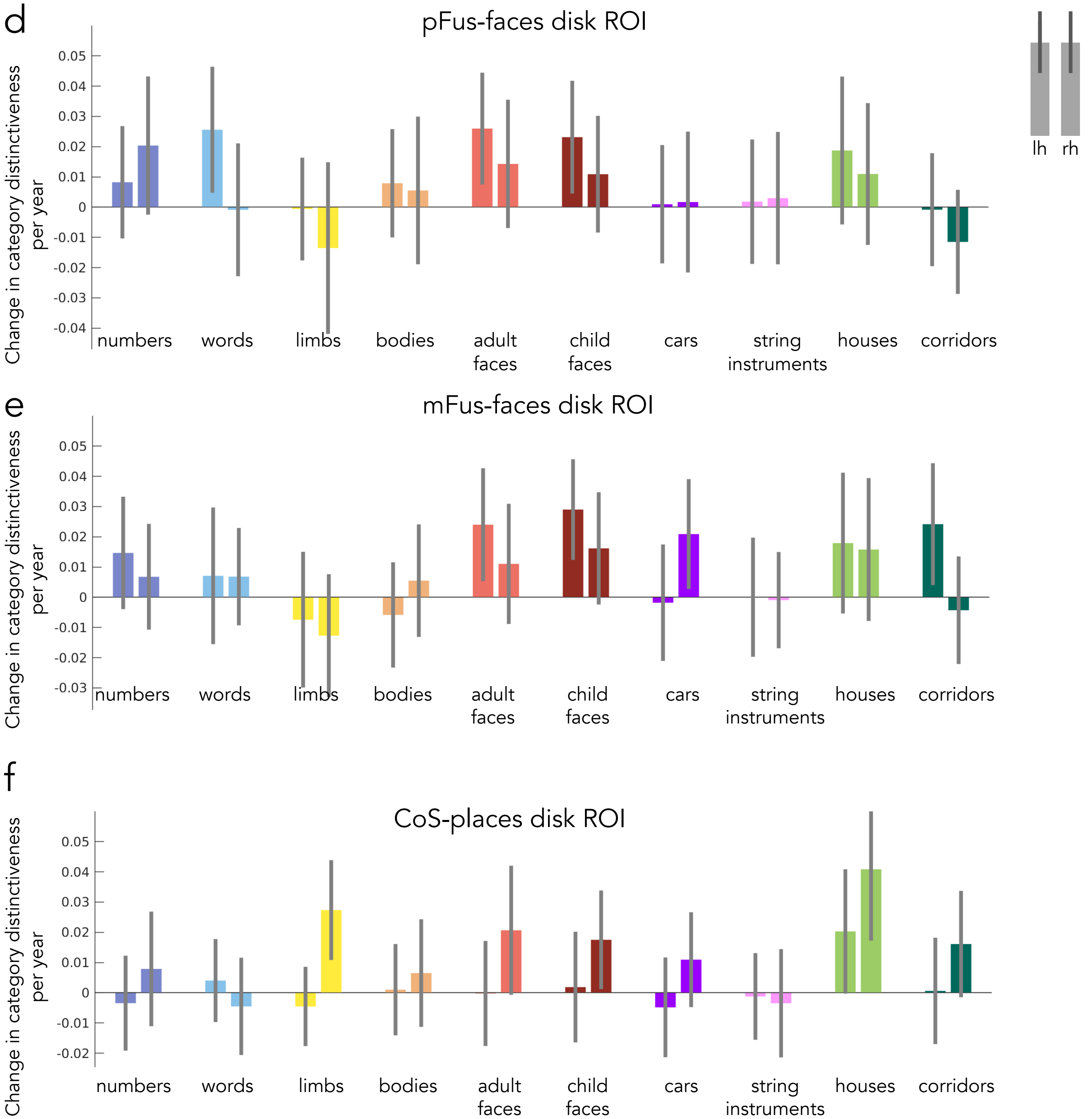

Fig S5. Development of distinctiveness in category-selective disk ROIs.
Changes in distinctiveness in disk ROIs centered on (A) pOTS-word (lh: n=28 participants, 125 sessions), (B) mOTS-word (lh: n=23 participants, 103 sessions), (C) OTS-limbs (lh: n=28 participants, 126 sessions; rh: n=28 participants, 126 sessions). (D) pFus-faces (lh: n=27 participants, 120 sessions; rh: n=22 participants, 98 sessions), (E) mFus-faces (lh: n=23 participants, 107 sessions; rh: n=24 participants, 106 sessions), (F) CoS-places (lh: n=29 participants, 128 sessions; rh=29 participants, 128 sessions). For pOTS-words and mOTS-words data is shown only for the left hemisphere due to low number of participants with ROIs in the right hemisphere. LMM slopes indicating change in distinctiveness per year in category-selective disk ROIs (LMM relating distinctiveness to age, with tSNR as an independent factor, and participant as random effect) for each category. *Error bars:* 95% confidence interval (CI). If the CI does not cross the y=0 line, the change in distinctiveness is significantly different than 0. Disk-ROIs (radius=10mm) were created for each category-selective ROI for each participant. To define the disk ROIs, ROIs of all sessions of that contrast in a given participant (i.e., word-selective ROI in session 1, 2 and 3) were used; ROIs had individually been defined on the respective participant's cortical surface. Then, the average center coordinates of the ROIs of all sessions belonging to that participant was computed and the disk ROI was centered on those mean coordinates. This approach was chosen as (i) it ensures that the voxels will show selectivity to a certain category, (ii) it ensures that the number of included voxels is constant across sessions of a participant, (iii) provides an independent definition of the ROI, and (iv) minimizes bias towards a particular session. Detailed statistics for this figure can be found in the data repository (see Methods, Data availability).

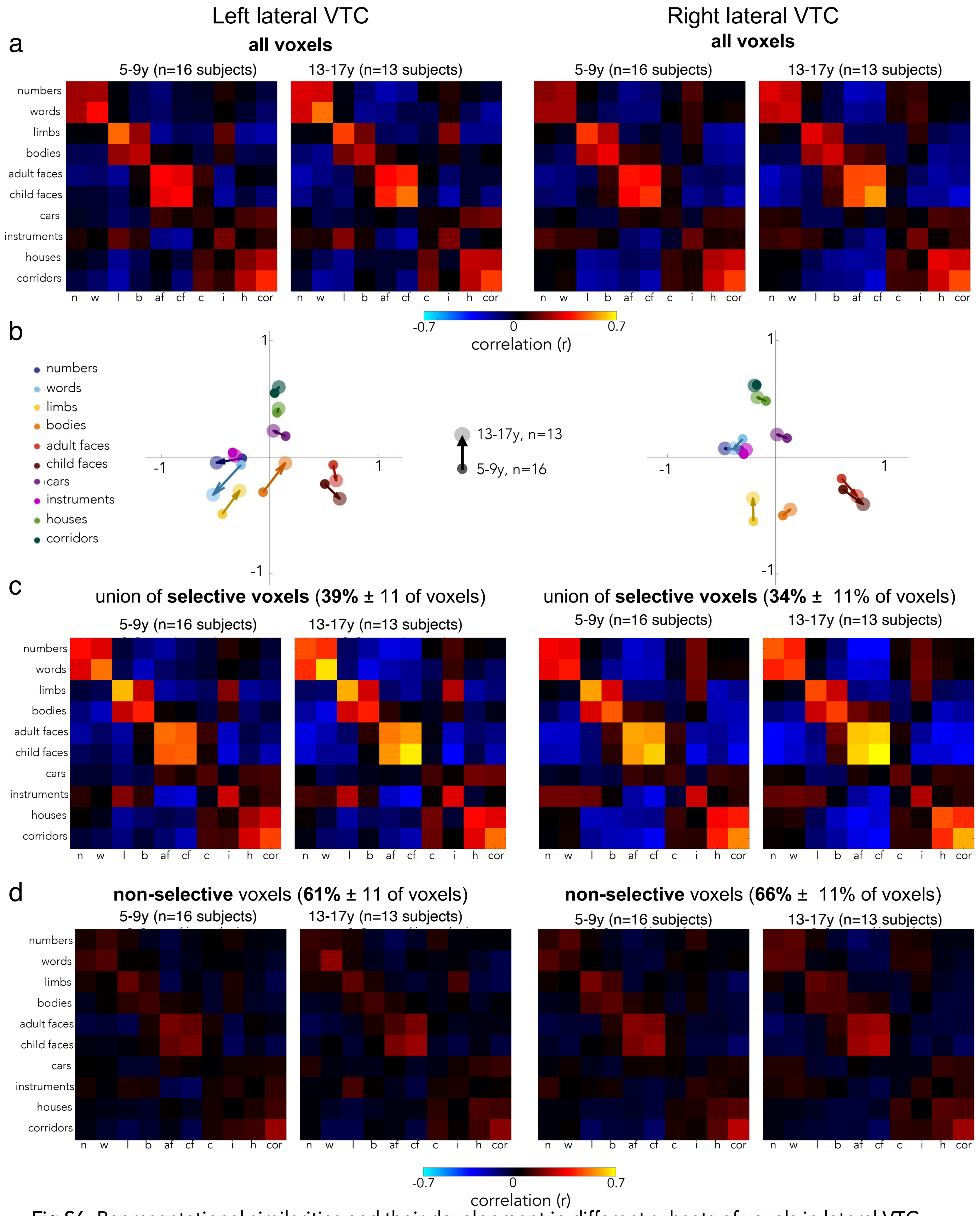

Fig S6. Representational similarities and their development in different subsets of voxels in lateral VTC. (A) Representational similarity matrices (RSM) of left and right lateral VTC for 5-9-year-olds and 13-17-year-olds. One session per child is included per RSM of each age group. (B) Multidimensional scaling (MDS) embeddings for the category representation for the same age groups: 5-9-year-olds (small circles) and 13-17-year-olds (larger circles). (C,D) Same as (A) but for different subsets of voxels; the union of the selective voxels (C) and the non-selective voxels (D). The color scale is the same across (A,C,D).

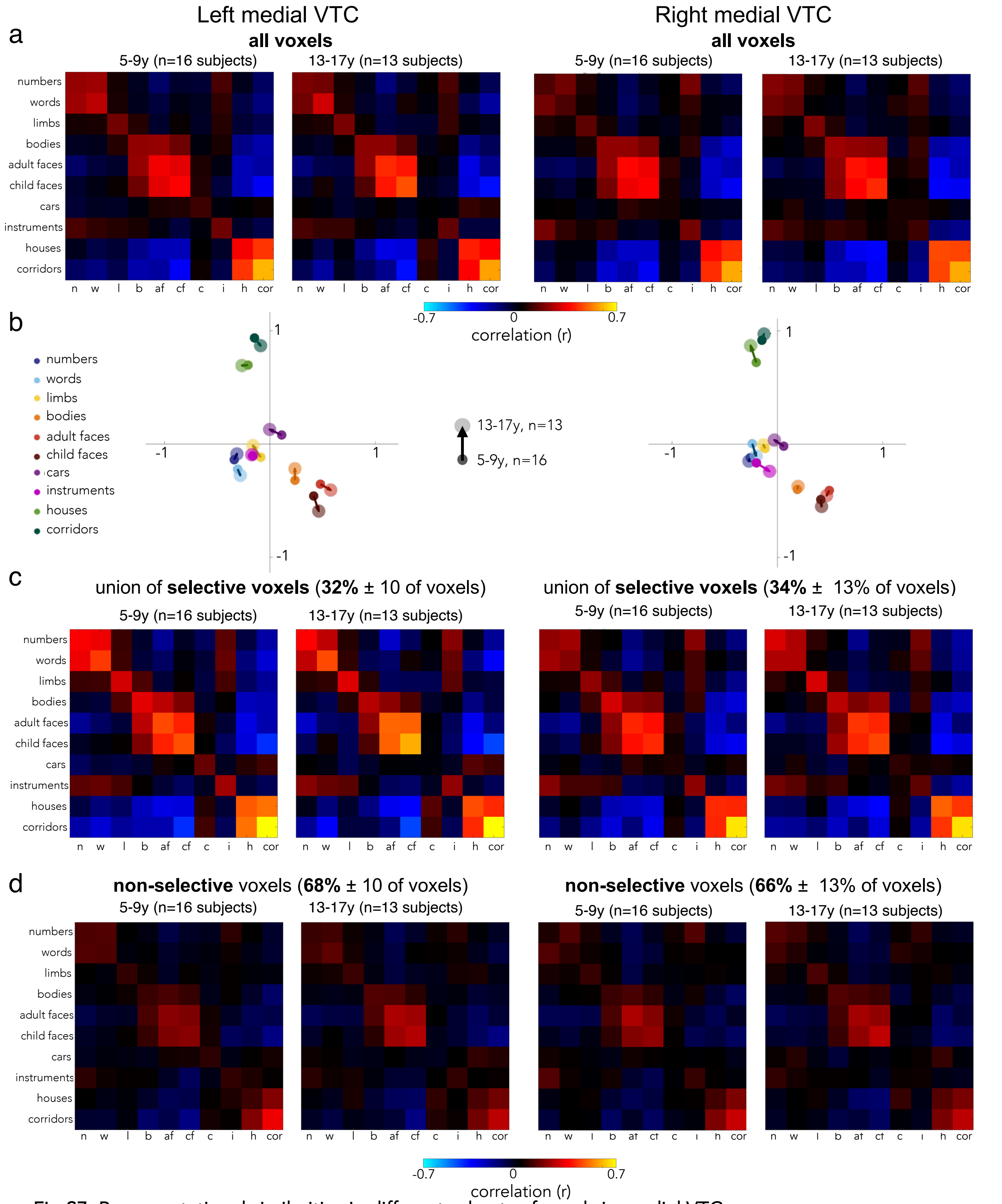

Fig S7. Representational similarities in different subsets of voxels in medial VTC.
(A) Representational similarity matrices (RSM) of left and right medial VTC for 5-9-year-olds and 13-17-year-olds. One session per child is included per RSM of each age group. (B) Multidimensional scaling (MDS) embeddings for the category representation for the same age groups: 5-9-year-olds (small circles) and 13-17-year-olds (larger circles). (C,D) Same as (A) but for different subsets of voxels; the union of the selective voxels (C) and the non-selective voxels (D). The color scale is the same across (A,C,D).

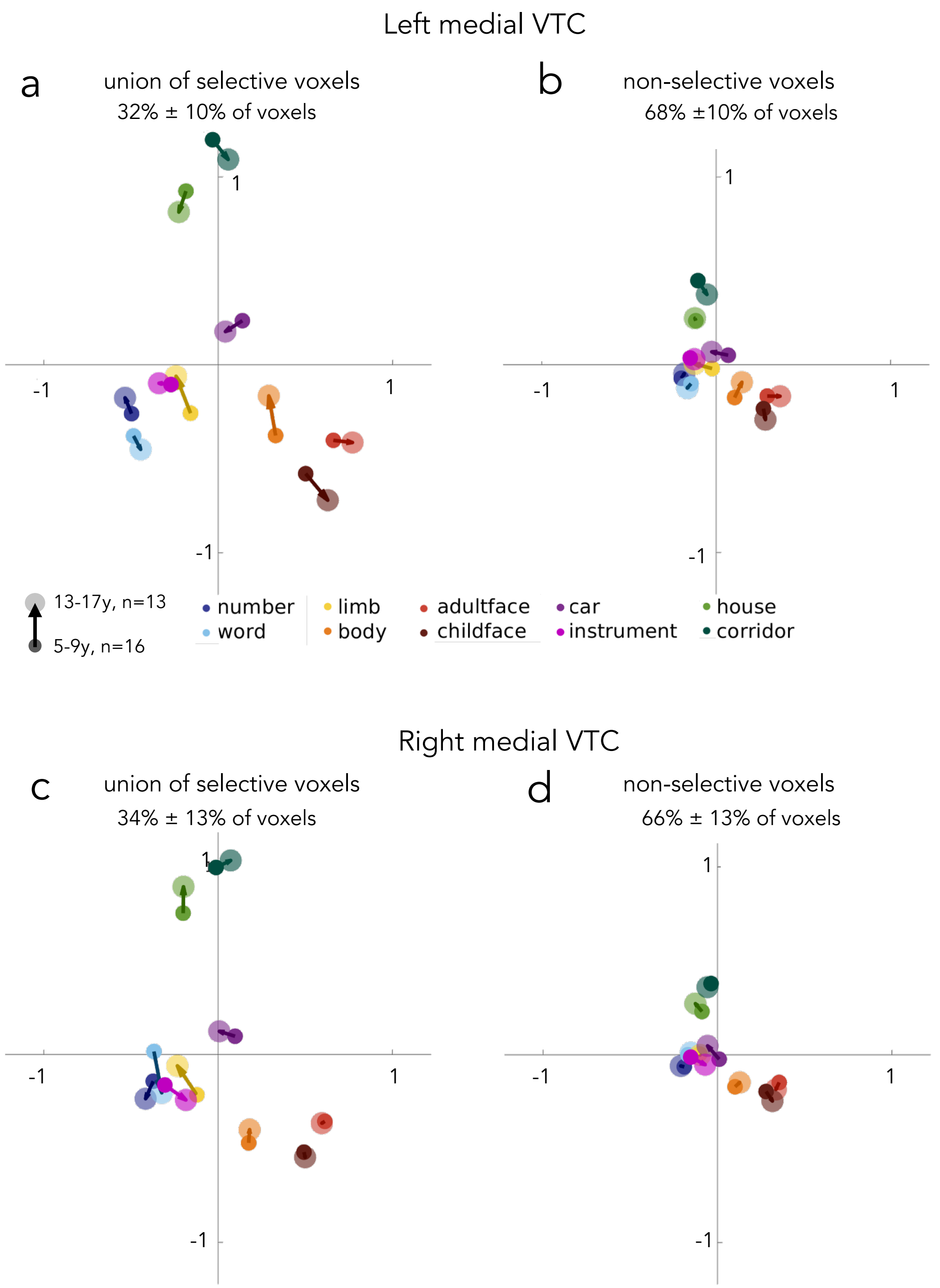

Fig S8. Representational space of the 10 categories in medial VTC. Multidimensional scaling (MDS) embeddings for the category representation in different subsets of voxels for two age groups: 5-9-year-olds (n=16 participants, small circles) and 13-17-year-olds (n=13 participants, larger circles). These age groups are used to illustrate the change in the representational space and comprise only two subsets of the whole sample. All statistics are run using the full sample (Fig. 2B). One session per child is included per MDS of each age group. (A,C) MDS embedding of the representational space across the union of selective voxels in left (A) and right (C) medial VTC. (B,D) MDS embedding of the representational space of the remainder, non-selective voxels of left (B) and right (D) medial VTC.

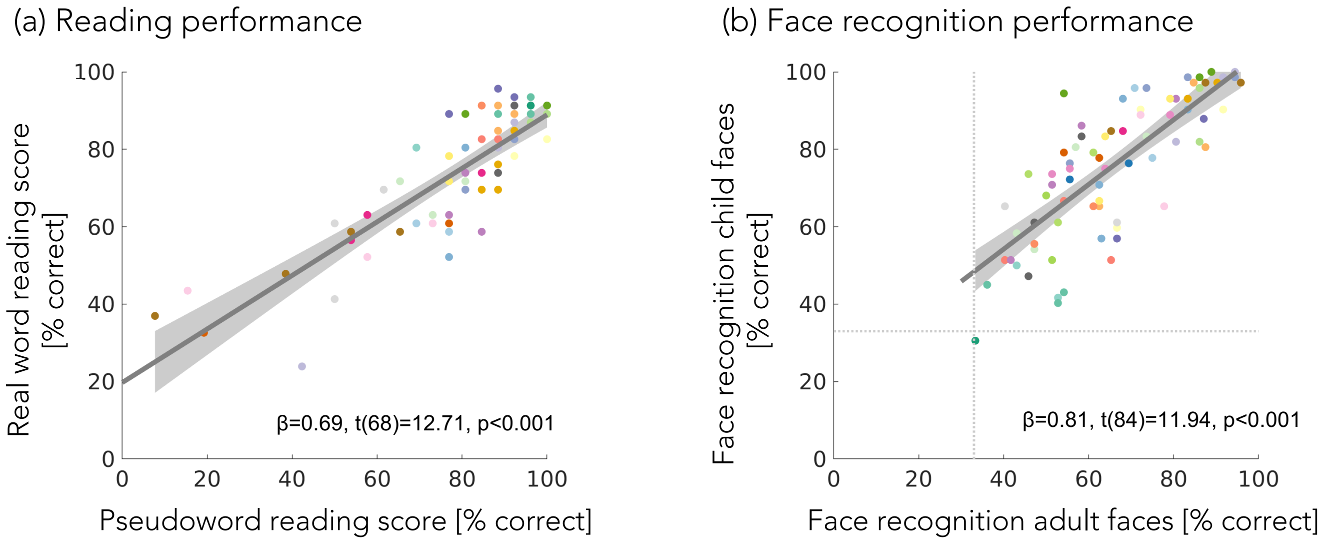

Fig S9. Reading and face recognition performance are reliable across tests

(A) Reading performance on pseudowords (x-axis) predicts reading performance on real words (y-axis), n=70 sessions, 26 children. (B) Face recognition performance in the Cambridge face recognition task (CFMT) with images of adults faces (x-axis) predicts performance on the CFMT test with images of child faces (y-axis), n=86 sessions, 28 children. Dashed line indicates chance level in the CFMT. Participants are coded by color. *Gray line:* Linear mixed model (LMM) prediction. *Shaded gray:* 95% confidence interval of the slope (CI).

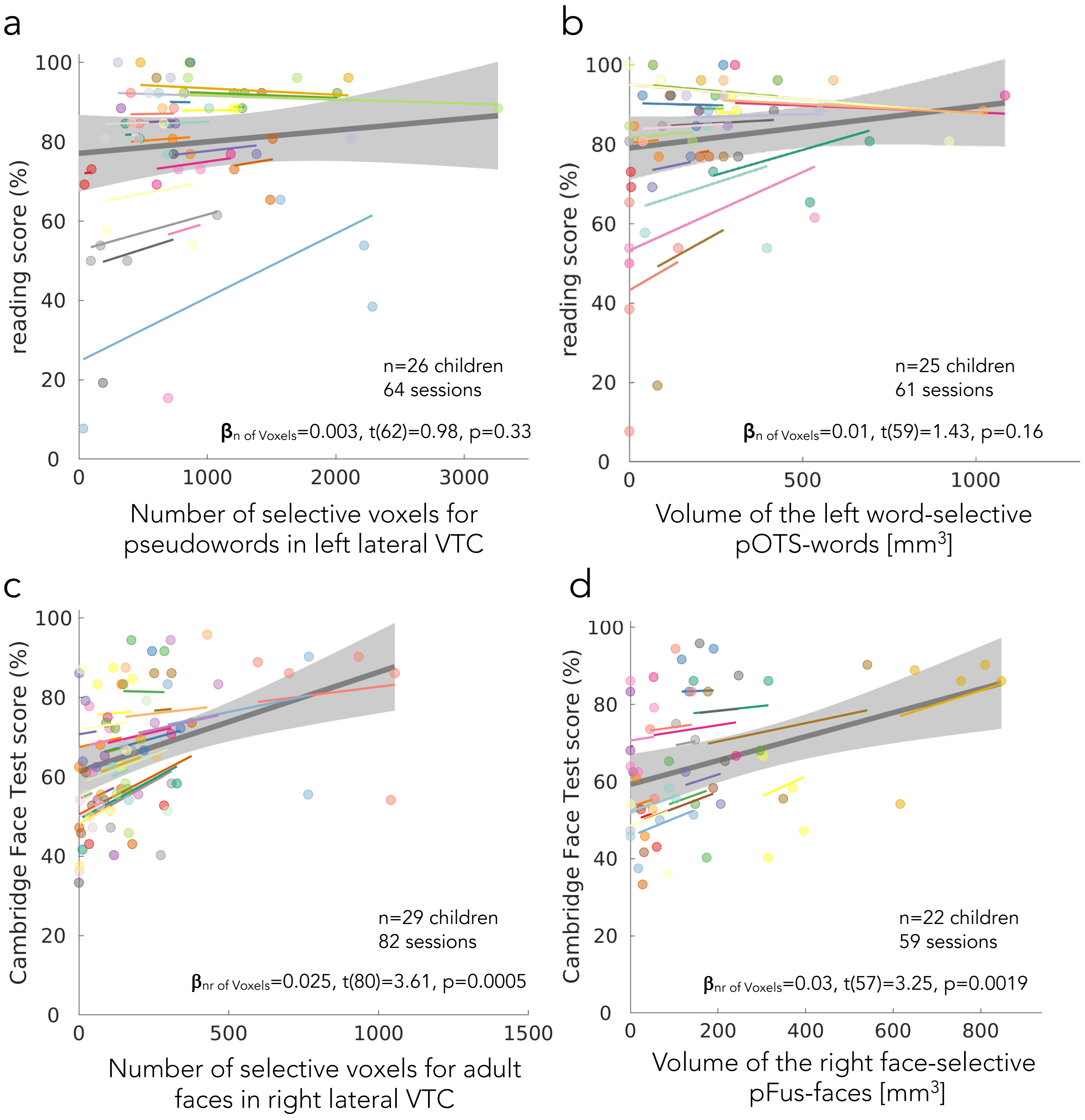

Fig S10. No significant link between behavior and the number of word- and face-selective voxels or the size of word- or face-selective ROIs after correcting for age. (A) Linear mixed model (LMM) with random slopes and intercepts relating reading performance of pseudowords (Woodcock Reading Mastery Test, WRMT) to the number of voxels selective to pseudowords in anatomically defined left lateral VTC ROIs. Selective voxels are defined by a t-value > 3 (contrast: pseudowords vs. all other categories except numbers). (B) Same as (A) but for volume of the left word-selective pOTS-words. (C) LMM with random slopes and intercepts relating performance in the Cambridge Face Memory Test (CFMT) to the number of voxels selective to adult faces in anatomically defined right lateral VTC ROIs. Selective voxels are defined by a t-value > 3 (contrast: adult faces vs. all other categories except child faces). The LMM shows a significant relation between face recognition performance and the number of selective voxels (model parameters and statistics in the figure). The effect of distinctiveness is no longer significant once age is added to the model: LMM with factors age and number of selective voxels: $\boldsymbol{\beta}_{\text{nr of Voxels}}=0.0065$, $t(79)=1.16$, $p=0.25$; $\boldsymbol{\beta}_{\text{age}}=3.78$, $t(79)=7.48$, $p<0.001$. (D) Same as (C) but for the number of voxels in the face-selective ROI pFus-faces in the right hemisphere. The LMM shows a significant effect of the number of selective voxels (see parameters indicated in the figure). This effect is no longer significant once age is added to the model: LMM with factors age and number of selective voxels: $\boldsymbol{\beta}_{\text{nr of Voxels}}=0.01$, $t(56)=1.22$, $p=0.23$; $\boldsymbol{\beta}_{\text{age}}=3.81$, $t(56)=5.94$ $p<0.001$. In all panels, model parameters and stats are indicated in the bottom. Each dot is a session; Dots are colored by participant. *Colored lines:* individual slopes and intercepts. *Thick gray line:* LMM prediction not including age in the model. *Shaded gray:* 95% CI.

Supplementary Table 1. Parameters and statistics of LMMs on changes in distinctiveness in lateral VTC ROIs per year. Related to Fig 1. FDR-corrected p-values are reported to adjust for multiple comparisons.

ROI	Contrast	parameter	β	CI	df	t	*p*	*p FDR*
Lh lateral VTC	Numbers	intercept	-0.12	-0.43; 0.19	125	-0.74	0.46	
		tSNR	0.004	0.001;0.007	125	2.70	0.008	
		age	0.011	-0.005;0.026	125	1.39	0.17	0.32
n=128 sessions	Words	intercept	0.08	-0.22;0.38	125	0.52	0.60	
		tSNR	0.002	-0.001;0.005	125	1.21	0.23	
		age	0.026	0.011;0.041	125	3.49	0.0007	0.0038
	Limbs	intercept	0.36	0.08;0.64	125	2.53	0.013	
		tSNR	0.003	0.0006;0.006	125	2.43	0.016	
		age	-0.006	-0.020;0.008	125	-0.88	0.38	0.54
	Headless bodies	intercept	0.11	-0.14;0.36	125	0.86	0.39	
		tSNR	0.0017	-0.0008;0.004	125	1.35	0.18	
		age	0.005	-0.007;0.018	125	0.89	0.38	0.54
	Adult faces	intercept	-0.009	-0.28;0.26	125	-0.07	0.95	
		tSNR	0.004	0.0012;0.0065	125	2.89	0.005	
		age	0.018	0.0049;0.031	125	2.72	0.008	0.019
	Child faces	intercept	-0.12	-0.4;0.16	125	-0.83	0.41	
		tSNR	0.005	0.002;0.007	125	3.16	0.002	
		age	0.029	0.015;0.043	125	4.06	8.49x10-5	0.0017
	Cars	intercept	-0.14	-0.41;0.13	125	-1.01	0.31	
		tSNR	0.002	-0.0007;0.005	125	1.51	0.13	
		age	4.4x10-5	-0.013;0.014	125	0.006	0.99	0.99
	Instruments	intercept	-0.31	-0.59; -0.04	125	-2.24	0.027	
		tSNR	0.006	0.003;0.009	125	4.39	2.36x10-5	
		age	0.001	-0.012;0.015	125	0.17	0.86	0.93
	Houses	intercept	-0.40	-0.73;-0.07	125	-2.42	0.017	
		tSNR	0.005	0.001;0.008	125	2.72	0.008	
		age	0.028	0.012;0.045	125	3.45	0.0008	0.0038
	Corridors	intercept	0.17	-0.10;0.45	125	1.26	0.21	
		tSNR	0.003	0.0003;0.006	125	2.18	0.03	
		age	0.008	-0.006;0.021	125	1.12	0.27	0.44
Rh lateral VTC	Numbers	intercept	-0.28	-0.55;-0.01	125	-2.08	0.0397	
		tSNR	0.005	0.002;0.007	125	3.20	0.002	
		age	0.023	0.009;0.037	125	3.36	0.001	0.004
n=128 sessions	Words	intercept	0.28	0.014;0.549	125	2.08	0.039	
		tSNR	-0.001	-0.004;0.001	125	-0.94	0.35	
		age	0.009	-0.004;0.023	125	1.36	0.18	0.32
	Limbs	intercept	0.31	0.015;0.598	125	2.08	0.039	
		tSNR	0.005	0.002;0.008	125	3.40	0.0009	
		age	-0.018	-0.033;-0.004	125	-2.45	0.016	0.035
	Headless bodies	intercept	0.03	-0.23;0.29	125	0.23	0.82	
		tSNR	0.005	0.002;0.008	125	3.66	0.0004	
		age	0.0017	-0.012;0.015	125	0.25	0.80	0.93
	Adult faces	intercept	-0.195	-0.47;0.077	125	-1.42	0.16	
		tSNR	0.008	0.005;0.011	125	5.35	4.02x10-7	
		age	0.0199	0.006;0.034	125	2.85	0.005	0.015
	Child faces	intercept	-0.09	-0.34;0.16	125	-0.70	0.48	
		tSNR	0.006	0.004;0.009	125	4.86	3.4x10-6	
		age	0.025	0.012;0.038	125	3.84	0.0002	0.002
	Cars	intercept	-0.05	-0.35;0.24	125	-0.35	0.73	
		tSNR	0.001	-0.002;0.004	125	0.77	0.44	
		age	-0.001	-0.016;0.014	125	-0.15	0.88	0.93
	Instruments	intercept	-0.04	-0.27;0.18	125	-0.39	0.697	
		tSNR	0.003	0.0003;0.005	125	2.23	0.027	
		age	0.0019	-0.009;0.013	125	0.34	0.74	0.92
	Houses	intercept	-0.31	-0.65;0.03	125	-1.79	0.08	
		tSNR	0.006	0.002;0.009	125	3.22	0.002	
		age	0.026	0.009;0.043	125	2.98	0.003	0.011
	Corridors	intercept	0.21	-0.027;0.454	125	1.76	0.08	
		tSNR	0.005	0.002;0.007	125	3.54	0.0006	
		age	0.003	-0.009;0.015	125	0.52	0.61	0.81

Supplementary Table 2. Parameters and statistics of LMMs on changes in distinctiveness in medial VTC ROIs per year. Related to Fig 1. FDR-corrected p-values are reported to adjust for multiple comparisons.

ROI	Contrast	parameter	β	CI	df	t	*p*	*pFDR*
Lh medial VTC	Numbers	intercept	-0.196	-0.45,0.06	125	-1.5	0.14	
		tSNR	0.007	0.004;0.01	125	4.28	3.62x10-5	
		age	0.0005	-0.011;0.012	125	0.09	0.93	0.98
n=128 sessions	Words	intercept	0.27	-0.05;0.59	125	1.65	0.10	
		tSNR	-0.0003	-0.004;0.004	125	-0.15	0.88	
		age	0.002	-0.012;0.016	125	0.29	0.77	0.98
	Limbs	intercept	-0.016	-0.32;0.29	125	-0.10	0.92	
		tSNR	0.003	-0.0002;0.007	125	1.89	0.06	
		age	-0.0019	-0.015;0.011	125	-0.29	0.78	0.98
	Headless bodies	intercept	-0.12	-0.38;0.14	125	-0.91	0.36	
		tSNR	0.005	0.002;0.008	125	3.34	0.001	
		age	0.0007	-0.01;0.01	125	0.13	0.9	0.98
	Adult faces	intercept	-0.013	-0.28;0.26	125	-0.098	0.92	
		tSNR	0.005	0.002;0.008	125	2.95	0.004	
		age	0.0186	0.007;0.03	125	3.048	0.0028	0.028
	Child faces	intercept	-0.098	-0.393;0.197	125	-0.66	0.51	
		tSNR	0.006	0.003;0.009	125	3.59	0.0005	
		age	0.0187	0.005;0.03	125	2.76	0.0067	0.034
	Cars	intercept	-0.25	-0.53;0.03	125	-1.76	0.08	
		tSNR	0.005	0.002;0.008	125	2.88	0.005	
		age	-0.004	-0.016;0.009	125	-0.60	0.55	0.98
	Instruments	intercept	-0.195	-0.41;0.023	125	-1.77	0.079	
		tSNR	0.005	0.0025;0.0078	125	3.82	0.0002	
		age	0.001	-0.008;0.0105	125	0.21	0.84	0.98
	Houses	intercept	-0.61	-0.98;-0.24	125	-3.27	0.001	
		tSNR	0.013	0.009;0.018	125	6.08	1.38x10-8	
		age	0.023	0.006;0.039	125	2.76	0.0067	0.034
	Corridors	intercept	0.13	-0.12;0.39	125	1.02	0.31	
		tSNR	0.01	0.007;0.013	125	6.57	1.19x10-9	
		age	-0.002	-0.013;0.009	125	-0.38	0.70	0.98
Rh medial VTC	Numbers	intercept	-0.433	-0.72;-0.14	125	-2.97	0.0035	
		tSNR	0.008	0.004;0.012	125	4.05	8.91x10-5	
		age	0.011	-0.0006;0.023	125	1.89	0.06	0.21
n=128 sessions	Words	intercept	0.45	0.15;0.76	125	2.95	0.0038	
		tSNR	-0.0058	-0.0099;-0.002	125	-2.80	0.0059	
		age	-0.0003	-0.013;0.012	125	-0.05	0.96	0.98
	Limbs	intercept	-0.29	-0.57;-0.008	125	-2.04	0.04	
		tSNR	0.007	0.0029;0.010	125	3.5	0.0006	
		age	0.006	-0.005;0.018	125	1.06	0.29	0.58
	Headless bodies	intercept	-0.22	-0.54;0.10	125	-1.35	0.18	
		tSNR	0.006	0.002;0.010	125	2.80	0.006	
		age	0.008	-0.006;0.022	125	1.19	0.24	0.52
	Adult faces	intercept	-0.036	-0.43;0.36	125	-0.18	0.86	
		tSNR	0.006	0.0009;0.011	125	2.31	0.02	
		age	0.013	-0.004;0.030	125	1.56	0.12	0.30
	Child faces	intercept	0.018	-0.33;0.36	125	0.10	0.92	
		tSNR	0.0045	-5.4x10-5;0.01	125	1.96	0.053	
		age	0.014	-0.0006;0.029	125	1.89	0.061	0.21
	Cars	intercept	-0.036	-0.39;0.32	125	-0.20	0.84	
		tSNR	0.001	-0.004;0.006	125	0.42	0.68	
		age	0.0002	-0.015;0.015	125	0.03	0.98	0.98
	Instruments	intercept	-0.18	-0.45;0.10	125	-1.26	0.21	
		tSNR	0.006	0.002;0.009	125	3.09	0.002	
		age	-0.0009	-0.01;0.01	125	-0.15	0.88	0.98
	Houses	intercept	-0.45	-0.86;-0.03	125	-2.14	0.035	
		tSNR	0.011	0.006;0.016	125	4.06	8.5x10-5	
		age	0.032	0.015;0.050	125	3.61	0.0004	0.009
	Corridors	intercept	0.14	-0.13;0.42	125	1.04	0.3	
		tSNR	0.009	0.005;0.012	125	4.96	2.2x10-6	
		age	0.010	-0.0015;0.022	125	1.72	0.087	0.25

Supplementary Table 3. Parameters and statistics of LMMs on changes in distinctiveness in the union of selective voxels in lateral VTC ROIs per year. Related to Fig 2. FDR-corrected p-values are reported to adjust for multiple comparisons.

ROI	Contrast	parameter	β	CI	df	t	*p*	*p FDR*
Lh lateral VTC	Numbers	intercept	0.0003	-0.34;0.34	125	0.002	0.998	
		tSNR	0.004	0.0007;0.008	125	2.397	0.018	
		age	0.016	0.0002;0.032	125	1.998	0.048	0.21
n=128 sessions	Words	intercept	0.35	0.082;0.624	125	2.57	0.011	
		tSNR	0.0005	-0.002;0.003	125	0.38	0.70	
		age	0.026	0.013;0.039	125	3.99	0.0001	0.002
	Limbs	intercept	0.55	0.278;0.82	125	4.00	0.0001	
		tSNR	0.002	-0.0007;0.005	125	1.47	0.15	
		age	0.0003	-0.01;0.01	125	0.054	0.96	0.96
	Headless bodies	intercept	0.25	-0.037;0.53	125	1.72	0.09	
		tSNR	0.002	-0.001;0.004	125	1.14	0.26	
		age	0.009	-0.005;0.022	125	1.31	0.19	0.50
	Adult faces	intercept	0.078	-0.21;0.36	125	0.54	0.59	
		tSNR	0.004	0.001;0.007	125	2.96	0.004	
		age	0.026	0.013;0.039	125	3.88	0.0002	0.002
	Child faces	intercept	0.11	-0.22;0.44	125	0.67	0.50	
		tSNR	0.003	0.0001;0.007	125	2.07	0.04	
		age	0.034	0.019;0.05	125	4.39	2.36x10-5	0.0009
	Cars	intercept	-0.009	-0.31;0.29	125	-0.06	0.95	
		tSNR	0.001	-0.002;0.004	125	0.85	0.4	
		age	-0.0004	-0.014;0.013	125	-0.062	0.95	0.96
	Instruments	intercept	-0.25	-0.60;0.09	125	-1.46	0.15	
		tSNR	0.006	0.003;0.01	125	3.62	0.0004	
		age	0.005	-0.011;0.021	125	0.64	0.52	0.79
	Houses	intercept	-0.30	-0.72;0.11	125	-1.45	0.15	
		tSNR	0.003	-0.0009;0.007	125	1.54	0.13	
		age	0.036	0.017;0.06	125	3.69	0.0003	0.003
	Corridors	intercept	0.35	0.034;0.67	125	2.19	0.03	
		tSNR	0.001	-0.002;0.004	125	0.85	0.4	
		age	0.01	-0.005;0.025	125	1.32	0.19	0.50
Rh lateral VTC	Numbers	intercept	0.049	-0.33;0.43	125	0.26	0.797	
		tSNR	0.0018	-0.002;0.006	125	0.90	0.37	
		age	0.027	0.0098;0.045	125	3.08	0.0025	0.017
n=128 sessions	Words	intercept	0.62	0.28;0.96	125	3.58	0.0005	
		tSNR	-0.003	-0.006;0.0005	125	-1.68	0.1	
		age	0.005	-0.01;0.02	125	0.65	0.52	0.79
	Limbs	intercept	0.53	0.21;0.86	125	3.25	0.001	
		tSNR	0.004	0.0002;0.007	125	2.11	0.037	
		age	-0.013	-0.028;0.002	125	-1.67	0.098	0.33
	Headless bodies	intercept	0.12	-0.19;0.43	125	0.75	0.46	
		tSNR	0.006	0.002;0.009	125	3.46	0.0007	
		age	0.007	-0.008;0.021	125	0.89	0.37	0.65
	Adult faces	intercept	0.33	0.03;0.62	125	2.20	0.03	
		tSNR	0.004	0.0007;0.007	125	2.44	0.016	
		age	0.02	0.007;0.034	125	2.95	0.004	0.02
	Child faces	intercept	0.51	0.24;0.77	125	3.78	0.0002	
		tSNR	0.0028	-1.4x10-6;0.006	125	1.98	0.05	
		age	0.017	0.0048;0.03	125	2.75	0.007	0.03
	Cars	intercept	-0.05	-0.43;0.33	125	-0.24	0.81	
		tSNR	0.0018	-0.002;0.006	125	0.88	0.38	
		age	0.0009	-0.016;0.018	125	0.1	0.92	0.96
	Instruments	intercept	0.13	-0.19;0.45	125	0.80	0.42	
		tSNR	0.0027	-0.0007;0.006	125	1.56	0.12	
		age	-0.004	-0.019;0.011	125	-0.49	0.63	0.79
	Houses	intercept	-0.05	-0.47;0.36	125	-0.26	0.80	
		tSNR	0.003	-0.0007;0.008	125	1.62	0.11	
		age	0.034	0.015;0.054	125	3.49	0.0007	0.005
	Corridors	intercept	0.47	0.16;0.78	125	3.03	0.003	
		tSNR	0.002	-0.001;0.005	125	1.33	0.19	
		age	0.009	-0.005;0.02	125	1.23	0.22	0.52

Supplementary Table 4. Parameters and statistics of LMMs on changes in distinctiveness in the non-selective voxels in lateral VTC ROIs per year. Related to Fig 2. FDR-corrected p-values are reported to adjust for multiple comparisons.

ROI	Contrast	parameter	β	CI	df	t	*p*	*p FDR*
Lh lateral VTC	Numbers	intercept	-0.05	-0.26;0.16	125	-0.44	0.66	
		tSNR	0.0009	-0.001;0.003	125	0.86	0.39	
		age	0.005	-0.006;0.017	125	0.96	0.34	0.64
n=128 sessions	Words	intercept	0.14	-0.06;0.34	125	1.37	0.17	
		tSNR	0.0004	-0.002;0.002	125	0.35	0.72	
		age	0.0005	-0.01;0.01	125	0.09	0.93	0.96
	Limbs	intercept	0.228	-0.012;0.47	125	1.88	0.06	
		tSNR	-0.0002	-0.003;0.002	125	-0.197	0.84	
		age	-0.004	-0.016;0.009	125	-0.56	0.57	0.79
	Headless bodies	intercept	0.066	-0.12;0.25	125	0.69	0.49	
		tSNR	-0.0002	-0.002;0.002	125	-0.18	0.86	
		age	0.004	-0.006;0.014	125	0.79	0.43	0.72
	Adult faces	intercept	0.2	-0.04;0.44	125	1.64	0.10	
		tSNR	0.0003	-0.002;0.003	125	0.22	0.83	
		age	-0.0009	-0.014;0.012	125	-0.14	0.89	0.96
	Child faces	intercept	0.14	-0.12;0.39	125	1.07	0.29	
		tSNR	4.98x10-5	-0.002;0.003	125	0.04	0.97	
		age	0.007	-0.007;0.02	125	0.97	0.33	0.64
	Cars	intercept	-0.12	-0.34;0.11	125	-1.03	0.31	
		tSNR	0.0015	-0.0008;0.004	125	1.31	0.19	
		age	-0.002	-0.01;0.01	125	-0.399	0.69	0.81
	Instruments	intercept	-0.096	-0.29;0.100	125	-0.97	0.33	
		tSNR	0.002	0.0004;0.0044	125	2.43	0.017	
		age	-0.003	-0.013;0.008	125	-0.51	0.61	0.79
	Houses	intercept	-0.11	-0.35;0.12	125	-0.95	0.34	
		tSNR	0.001	-0.001;0.004	125	1.08	0.28	
		age	0.008	-0.004;0.02	125	1.31	0.19	0.50
	Corridors	intercept	0.24	0.018;0.469	125	2.13	0.035	
		tSNR	0.0005	-0.002;0.003	125	0.442	0.66	
		age	-0.003	-0.015;0.009	125	-0.48	0.63	0.79
Rh lateral VTC	Numbers	intercept	-0.17	-0.34;-0.005	125	-2.04	0.04	
		tSNR	0.002	0.0007;0.004	125	2.75	0.007	
		age	0.009	-0.0004;0.019	125	1.9	0.06	0.24
n=128 sessions	Words	intercept	0.14	-0.05;0.32	125	1.47	0.14	
		tSNR	-0.001	-0.003;0.0006	125	-1.37	0.17	
		age	0.006	-0.004;0.016	125	1.12	0.27	0.59
	Limbs	intercept	0.07	-0.13;0.27	125	0.7	0.49	
		tSNR	0.002	3.4x10-5;0.004	125	2.01	0.046	
		age	-0.005	-0.016;0.006	125	-0.89	0.37	0.65
	Headless bodies	intercept	0.05	-0.12;0.22	125	0.60	0.55	
		tSNR	0.001	-0.0005;0.003	125	1.36	0.18	
		age	0.002	-0.007;0.01	125	0.42	0.68	0.81
	Adult faces	intercept	-0.016	-0.23;0.20	125	-0.14	0.89	
		tSNR	0.003	0.001;0.006	125	3.02	0.003	
		age	0.004	-0.009;0.016	125	0.57	0.57	0.79
	Child faces	intercept	0.14	-0.07;0.36	125	1.3	0.195	
		tSNR	0.0009	-0.001;0.003	125	0.85	0.4	
		age	0.006	-0.006;0.018	125	0.98	0.33	0.64
	Cars	intercept	-0.01	-0.21;0.18	125	-0.11	0.91	
		tSNR	0.0002	-0.002;0.002	125	0.16	0.87	
		age	-0.003	-0.014;0.008	125	-0.52	0.60	0.79
	Instruments	intercept	0.037	-0.14;0.21	125	0.42	0.67	
		tSNR	0.0004	-0.001;0.002	125	0.41	0.68	
		age	0.0002	-0.01;0.01	125	0.049	0.96	0.96
	Houses	intercept	-0.13	-0.35;0.09	125	-1.16	0.25	
		tSNR	0.003	0.0003;0.005	125	2.23	0.027	
		age	0.008	-0.004;0.02	125	1.28	0.20	0.50
	Corridors	intercept	0.23	0.07;0.4	125	2.78	0.006	
		tSNR	0.002	0.0007;0.004	125	2.79	0.006	
		age	-0.008	-0.018;0.0009	125	-1.79	0.08	0.28

Supplementary Table 5. Parameters and statistics of LMMs on changes in distinctiveness in the union of selective voxels in medial VTC ROIs per year. Related to Fig 2. FDR-corrected p-values are reported to adjust for multiple comparisons.

ROI	Contrast	parameter	β	CI	df	t	*p*	*p FDR*
Lh medial VTC	Numbers	intercept	0.048	-0.30;0.396	125	0.27	0.78	
		tSNR	0.005	0.0006;0.009	125	2.26	0.025	
		age	0.0066	-0.009;0.023	125	0.81	0.42	0.84
n=128 sessions	Words	intercept	0.67	0.27;1.07	125	3.33	0.001	
		tSNR	-0.004	-0.009;0.001	125	-1.52	0.13	
		age	0.001	-0.018;0.02	125	0.11	0.91	0.98
	Limbs	intercept	0.18	-0.22;0.58	125	0.91	0.37	
		tSNR	0.002	-0.003;0.007	125	0.88	0.38	
		age	-0.0004	-0.019;0.018	125	-0.044	0.96	0.99
	Headless bodies	intercept	0.12	-0.24;0.49	125	0.68	0.496	
		tSNR	0.004	-9.x10-5;0.009	125	1.94	0.055	
		age	-0.007	-0.023;0.01	125	-0.77	0.44	0.84
	Adult faces	intercept	0.28	-0.064;0.64	125	1.61	0.11	
		tSNR	0.0028	-0.001;0.007	125	1.35	0.18	
		age	0.015	-0.0014;0.032	125	1.81	0.07	0.46
	Child faces	intercept	0.26	-0.11;0.63	125	1.40	0.16	
		tSNR	0.003	-0.0007;0.008	125	1.65	0.10	
		age	0.0155	-0.002;0.033	125	1.74	0.085	0.46
	Cars	intercept	-0.15	-0.53;0.23	125	-0.77	0.44	
		tSNR	0.006	0.002;0.011	125	2.67	0.008	
		age	-0.015	-0.03;0.002	125	-1.70	0.09	0.46
	Instruments	intercept	-0.037	-0.33;0.26	125	-0.25	0.80	
		tSNR	0.005	0.0009;0.008	125	2.47	0.015	
		age	-0.0035	-0.017;0.01	125	-0.51	0.61	0.94
	Houses	intercept	-0.09	-0.49;0.30	125	-0.46	0.64	
		tSNR	0.01	0.006;0.015	125	4.33	2.98x10-5	
		age	0.007	-0.011;0.026	125	0.77	0.44	0.84
	Corridors	intercept	0.66	0.40;0.92	125	5.07	1.39x10-6	
		tSNR	0.005	0.0019;0.008	125	3.17	0.0019	
		age	-0.003	-0.015;0.009	125	-0.56	0.57	0.94
Rh medial VTC	Numbers	intercept	-0.349	-0.72;0.03	125	-1.845	0.067	
		tSNR	0.008	0.003;0.01	125	3.36	0.001	
		age	0.014	-0.003;0.03	125	1.64	0.10	0.46
n=128 sessions	Words	intercept	0.55	0.12;0.97	125	2.53	0.013	
		tSNR	-0.007	-0.012;-0.0009	125	-2.29	0.024	
		age	0.0016	-0.018;0.02	125	0.16	0.87	0.98
	Limbs	intercept	-0.27	-0.63;0.09	125	-1.48	0.14	
		tSNR	0.007	0.002;0.012	125	2.93	0.004	
		age	0.013	-0.003;0.029	125	1.59	0.11	0.46
	Headless bodies	intercept	-0.16	-0.55;0.24	125	-0.78	0.43	
		tSNR	0.007	0.002;0.012	125	2.81	0.006	
		age	0.0028	-0.015;0.021	125	0.311	0.76	0.94
	Adult faces	intercept	0.077	-0.341;0.495	125	0.36	0.72	
		tSNR	0.005	-0.0003;0.011	125	1.86	0.065	
		age	0.014	-0.005;0.033	125	1.45	0.15	0.52
	Child faces	intercept	0.047	-0.34;0.43	125	0.24	0.81	
		tSNR	0.005	0.0003;0.01	125	2.12	0.036	
		age	0.013	-0.005;0.03	125	1.43	0.156	0.52
	Cars	intercept	-0.19	-0.611;0.23	125	-0.91	0.37	
		tSNR	0.003	-0.002;0.009	125	1.21	0.23	
		age	0.004	-0.015;0.023	125	0.41	0.69	0.94
	Instruments	intercept	0.24	-0.09;0.58	125	1.43	0.15	
		tSNR	-0.0004	-0.005;0.004	125	-0.19	0.85	
		age	-0.002	-0.017;0.013	125	-0.28	0.78	0.94
	Houses	intercept	-0.26	-0.66;0.15	125	-1.26	0.21	
		tSNR	0.009	0.004;0.015	125	3.38	0.00097	
		age	0.028	0.0097;0.046	125	3.03	0.003	0.12
	Corridors	intercept	0.599	0.296;0.902	125	3.91	0.0001	
		tSNR	0.003	-0.0008;0.007	125	1.58	0.12	
		age	0.0039	-0.01;0.018	125	0.56	0.58	0.94

Supplementary Table 6. Parameters and statistics of LMMs on changes in distinctiveness in the non-selective voxels in medial VTC ROIs per year. Related to Fig 2. FDR-corrected p-values are reported to adjust for multiple comparisons.

ROI	Contrast	parameter	β	CI	df	t	*p*	*p FDR*
Lh medial VTC	Numbers	intercept	-0.14	-0.34;0.055	125	-1.43	0.156	
		tSNR	0.004	0.002;0.007	125	3.59	0.0005	
		age	-0.0004	-0.009;0.008	125	-0.08	0.93	0.98
n=128 sessions	Words	intercept	0.159	-0.056;0.374	125	1.46	0.146	
		tSNR	-0.0001	-0.003;0.002	125	-0.099	0.92	
		age	-0.0029	-0.013;0.007	125	-0.599	0.55	0.94
	Limbs	intercept	-0.11	-0.32;0.11	125	-0.98	0.33	
		tSNR	0.003	0.0004;0.006	125	2.31	0.022	
		age	0.0005	-0.009;0.010	125	0.11	0.91	0.98
	Headless bodies	intercept	-0.06	-0.24;0.12	125	-0.67	0.50	
		tSNR	0.002	0.0003;0.005	125	2.24	0.027	
		age	0.0012	-0.007;0.009	125	0.29	0.77	0.94
	Adult faces	intercept	-0.099	-0.32;0.12	125	-0.88	0.38	
		tSNR	0.0037	0.001;0.006	125	2.88	0.0046	
		age	0.012	0.002;0.022	125	2.41	0.018	0.35
	Child faces	intercept	-0.19	-0.46;0.08	125	-1.39	0.17	
		tSNR	0.005	0.002;0.008	125	3.14	0.002	
		age	0.0128	0.0005;0.025	125	2.06	0.041	0.41
	Cars	intercept	-0.14	-0.38;0.11	125	-1.09	0.28	
		tSNR	0.003	-3.2x10-5;0.006	125	1.96	0.053	
		age	-0.006	-0.017;0.005	125	-1.05	0.29	0.69
	Instruments	intercept	-0.06	-0.26;0.13	125	-0.65	0.51	
		tSNR	0.002	-0.0004;0.004	125	1.63	0.11	
		age	0.0008	-0.008;0.009	125	0.18	0.86	0.98
	Houses	intercept	-0.30	-0.601;-0.003	125	-1.999	0.048	
		tSNR	0.005	0.0017;0.009	125	2.95	0.0038	
		age	0.007	-0.006;0.021	125	1.1	0.29	0.69
	Corridors	intercept	0.012	-0.256;0.28	125	0.09	0.93	
		tSNR	0.007	0.004;0.01	125	4.5	1.6x10-5	
		age	-0.01	-0.022;0.002	125	-1.67	0.098	0.46
Rh medial VTC	Numbers	intercept	-0.39	-0.63;-0.15	125	-3.19	0.002	
		tSNR	0.006	0.0027;0.0092	125	3.63	0.0004	
		age	0.011	0.0009;0.021	125	2.16	0.033	0.41
n=128 sessions	Words	intercept	0.29	0.05;0.53	125	2.43	0.017	
		tSNR	-0.004	-0.007;-0.0004	125	-2.21	0.029	
		age	-0.0023	-0.012;0.008	125	-0.45	0.66	0.94
	Limbs	intercept	-0.18	-0.42;0.053	125	-1.53	0.13	
		tSNR	0.0045	0.0013;0.008	125	2.76	0.007	
		age	8.2x10-5	-0.0099;0.010	125	0.016	0.99	0.99
	Headless bodies	intercept	0.019	-0.21;0.247	125	0.16	0.87	
		tSNR	0.0006	-0.0025;0.0036	125	0.375	0.71	
		age	0.004	-0.005;0.014	125	0.86	0.39	0.84
	Adult faces	intercept	-0.04	-0.33;0.25	125	-0.28	0.78	
		tSNR	0.0039	0.0001;0.008	125	2.06	0.042	
		age	0.0067	-0.0059;0.019	125	1.05	0.29	0.69
	Child faces	intercept	0.044	-0.25;0.34	125	0.299	0.77	
		tSNR	0.0017	-0.002;0.006	125	0.86	0.39	
		age	0.009	-0.004;0.021	125	1.37	0.17	0.53
	Cars	intercept	-0.029	-0.30;0.25	125	-0.21	0.84	
		tSNR	-0.0001	-0.0038;0.0036	125	-0.06	0.96	
		age	0.0019	-0.0097;0.013	125	0.32	0.75	0.94
	Instruments	intercept	-0.21	-0.43;0.002	125	-1.96	0.052	
		tSNR	0.005	0.002;0.008	125	3.42	0.0008	
		age	-0.002	-0.011;0.007	125	-0.46	0.65	0.94
	Houses	intercept	-0.27	-0.56;0.02	125	-1.84	0.068	
		tSNR	0.005	0.001;0.009	125	2.66	0.009	
		age	0.0072	-0.005;0.0197	125	1.15	0.25	0.69
	Corridors	intercept	0.195	-0.05;0.44	125	1.56	0.12	
		tSNR	0.002	-0.001;0.005	125	1.35	0.18	
		age	-0.0015	-0.0124;0.009	125	-0.28	0.78	0.94
