# Supplementary material for: Longitudinal development of category representations in ventral temporal cortex predicts word and face recognition
Source: Nat Commun. 2023 Dec 4;14:8010. doi: 10.1038/s41467-023-43146-w (PMC10696026; doi:10.1038/s41467-023-43146-w)
Supplement: Supplementary file 3 — Description of Additional Supplementary Files [file 41467_2023_43146_MOESM3_ESM.pdf]

Description of Additional Supplementary Files

File Name: Supplementary Movie 1

Description: **Development of the representational space of the 10 categories in the union of the selective voxels in left and right lateral VTC.** The movie shows how the multidimensional scaling (MDS) embeddings for the category representation develop from 5-9-year-olds (n=16 participants) to 13-17-year-olds (n=13 participants). The MDS embeddings are based on average RSMs of children in the two age groups. The movie shows the interpolation from the MDS embedding of 5-9-year-olds to that of 13-17-year-olds. Left side of the movie: change of MDS embeddings over the union of selective voxels in left lateral VTC. Right side of the movie: change of MDS embeddings over the union of selective voxels in right lateral VTC. The colors of the circles correspond to the 10 categories (Fig. 3). Dark blue: numbers; light blue: words; yellow: limbs, orange: bodies; light red: adult faces; dark red: child faces; purple: cars; pink: instruments; light green: houses; dark green: corridors.
