# Supplementary material for: Longitudinal development of category representations in ventral temporal cortex predicts word and face recognition
Source: Nat Commun. 2023 Dec 4;14:8010. doi: 10.1038/s41467-023-43146-w (PMC10696026; doi:10.1038/s41467-023-43146-w)
Supplement: Supplementary file 5 — Reporting Summary [file 41467_2023_43146_MOESM5_ESM.pdf]

nature portfolio

Corresponding author(s): Marisa Nordt

Last updated by author(s): Sep 20, 2023

Reporting Summary

Nature Portfolio wishes to improve the reproducibility of the work that we publish. This form provides structure for consistency and transparency in reporting. For further information on Nature Portfolio policies, see our Editorial Policies and the Editorial Policy Checklist.

Statistics

For all statistical analyses, confirm that the following items are present in the figure legend, table legend, main text, or Methods section.

n/a	Confirmed	
☐	☒	The exact sample size (*n*) for each experimental group/condition, given as a discrete number and unit of measurement
☐	☒	A statement on whether measurements were taken from distinct samples or whether the same sample was measured repeatedly
☐	☒	The statistical test(s) used AND whether they are one- or two-sided<br>*Only common tests should be described solely by name; describe more complex techniques in the Methods section.*
☐	☒	A description of all covariates tested
☐	☒	A description of any assumptions or corrections, such as tests of normality and adjustment for multiple comparisons
☐	☒	A full description of the statistical parameters including central tendency (e.g. means) or other basic estimates (e.g. regression coefficient) AND variation (e.g. standard deviation) or associated estimates of uncertainty (e.g. confidence intervals)
☐	☒	For null hypothesis testing, the test statistic (e.g. *F*, *t*, *r*) with confidence intervals, effect sizes, degrees of freedom and *P* value noted<br>*Give P values as exact values whenever suitable.*
☒	☐	For Bayesian analysis, information on the choice of priors and Markov chain Monte Carlo settings
☐	☒	For hierarchical and complex designs, identification of the appropriate level for tests and full reporting of outcomes
☒	☐	Estimates of effect sizes (e.g. Cohen's *d*, Pearson's *r*), indicating how they were calculated

Our web collection on statistics for biologists contains articles on many of the points above.

Software and code

Policy information about availability of computer code

Data collection	Code to the 10-category experiment: https://github.com/VPNL/fLoc
Data analysis	Data analysis was performed in MATLAB version 2017b (The MathWorks, Inc.) and using the open source mrVista software package (https://github.com/vistalab/vistasoft/wiki/mrVista, version 2.1). Preprocessing of the functional data was performed using the code provided in: https://github.com/VPNL/fLoc. Swarm plots in Fig. 4 were created using MATLAB version 2020b. Quantitative whole brain images of each child and timepoint were processed with the mrQ pipeline (https://github.com/mezera/mrQ). Each participant's individual brain anatomical template was generated using the FreeSurfer Longitudinal pipeline (https://surfer.nmr.mgh.harvard.edu/fswiki/LongitudinalProcessing) using FreeSurfer version 6. Original code to generate the main figures is available at https://github.com/VPNL/distributedVTCDevelopment and doi.org/10.5281/zenodo.8366779 (Nordt, M. et al. Longitudinal development of category representations in ventral temporal cortex predicts word and face recognition. VPNL/distributedVTCDevelopment (2023) doi:10.5281/zenodo.8366779.)

For manuscripts utilizing custom algorithms or software that are central to the research but not yet described in published literature, software must be made available to editors and reviewers. We strongly encourage code deposition in a community repository (e.g. GitHub). See the Nature Portfolio guidelines for submitting code & software for further information.

Data

Policy information about availability of data

All manuscripts must include a data availability statement. This statement should provide the following information, where applicable:

- Accession codes, unique identifiers, or web links for publicly available datasets
- A description of any restrictions on data availability
- For clinical datasets or third party data, please ensure that the statement adheres to our policy

The processed data required to generate the main and supplemental figures are available at: https://github.com/VPNL/distributedVTCDevelopment and doi.org/10.5281/zenodo.8366779. The data availability section is provided in the manuscript.

Research involving human participants, their data, or biological material

Policy information about studies with human participants or human data. See also policy information about sex, gender (identity/presentation), and sexual orientation and race, ethnicity and racism.

Reporting on sex and gender	In this study we report data of a non-representative sample of 29 children (18 female, 11 male). While we aimed to recruit a balanced sample with regard to gender, the final sample was not completely balanced in this regard. Gender was not considered in the design of the current study. Gender was determined based on self-reporting. The information will be reported with the source data. No sex and gender based analyses were performed as the final sample was not completely balanced according to these factors and the limited sample size does not enable deriving meaningful conclusions regarding sex and gender effects.
Reporting on race, ethnicity, or other socially relevant groupings	We report the race and ethnicity of our participants in the participants subsection of the Methods section. In brief,the diversity of the participants in this study reflects the makeup of the region around Palo Alto (CA): 62.5% of children were Caucasian, 20% were Asian, 5% were Native Hawaiian, 5% were Hispanic, and 7.5% were multiracial or from other racial/ethnic groups. This data was assessed by self-report of the participants.
Population characteristics	See below.
Recruitment	Children were recruited by means of convenience sampling from schools in and around Palo Alto, CA via advertisements in local schools and on campus. The sample of participants reflects the makeup of the region around Palo Alto (CA), a region with a high percentage of residents with a bachelor's degree or higher. It is possible that the characteristics of this sample may influence the results.
Ethics oversight	This study was approved by the Institutional Review Board of Stanford University

Note that full information on the approval of the study protocol must also be provided in the manuscript.

Field-specific reporting

Please select the one below that is the best fit for your research. If you are not sure, read the appropriate sections before making your selection.

☐ Life sciences ☒ Behavioural & social sciences ☐ Ecological, evolutionary & environmental sciences

For a reference copy of the document with all sections, see nature.com/documents/nr-reporting-summary-flat.pdf

Behavioural & social sciences study design

All studies must disclose on these points even when the disclosure is negative.

Study description	This is a quantitative longitudinal study.
Research sample	Children (18 female, 11 male) aged 5-12 years with normal or corrected-to-normal vision were recruited for this study (mean=9.19, SD=2.13). This age range was selected for two reasons: First, face recognition and reading, the two behavioral measures assessed in this study, improve during this age range. Second, prior studies investigating the functional development of VTC have shown development in this age range. The number of participants in this study is within the ballpark of fMRI studies of the development of visual cortex, where the typical number of participants is between 10-20 participants.<br><br>Children were recruited in and around Palo Alto, CA. The diversity of the participants reflects the makeup of the region: 62.5% of children were Caucasian, 20% were Asian, 5% were Native Hawaiian, 5% were Hispanic, and 7.5% were multiracial or from other racial/ethnic groups.
Sampling strategy	Participants were recruited from local schools in and around Palo Alto, CA. Our number of participants is similar or exceeds previous cross-sectional publications and is larger than previous longitudinal studies on VTC development. To our knowledge except for our prior publication (Nordt NHB 2021) this is the largest dataset containing fMRI data of the development of ventral temporal cortex and includes 128 sessions collected on different days. No statistical method was used to predetermine sample size.

Data collection	Neuroimaging data: Prior to the fMRI experiment, participants were trained in an MRI simulator and acclimated to the MRI environment. Participants participated in functional and structural magnetic resonance imaging (MRI) experiments, which were conducted over several sessions. MRI data are securely transferred from the MR scanner directly to a data management system. There were always at least two researchers present for MRI sessions. In some cases parents stayed in the MRI control room during the MRI session. Behavioral data: Face recognition and reading tests were typically completed after one of the scanning sessions. Face recognition tests were administered on a computer. Reading tests were assessed by a native English speaker. All participants performed the same experiments, researchers were not blinded to the experimental conditions, and the same experiment was repeated across sessions.
Timing	The duration of participation in this study varied across participants. Each child participated in at least 2 and up to 10 fMRI sessions (mean=4.41, SD=1.92) with the time interval between the first and last fMRI scan ranging from 10 months to 5 years (mean=45 months, SD=18 months). Measurements were conducted between May 2014 and November 2019.
Data exclusions	We collected data from 40 (26 female) children. Data from 4 children were excluded because they dropped out of the study after participating only once, and thus did not provide longitudinal data. Data from 7 children were excluded because their data did not pass inclusion criteria (see below). In the remaining 29 children, 29 functional sessions were excluded due to motion, 1 session due to a technical error during acquisition, and 1 session due to aliasing artifacts during acquisition. Therefore, data from 128 functional sessions from 29 children (18 female) are reported in this study.<br><br>Inclusion criteria were as follows: In each functional session children participated in three runs of the 10-category experiment. Criteria for inclusion of data were: (i) at least 2 runs per session having within-run motion < 2 voxels and between-run motion < 3 voxels, and (ii) at least two fMRI sessions at least six months apart.<br>Because only two of the three runs survived motion quality thresholds for several fMRI sessions, analyses include two runs per child per session to ensure equal amounts of data across participants and sessions.<br><br>For analyses that relate fMRI data to behavioral data, behavioral datasets (face recognition and reading tests) were included in the analysis if the time between acquisition of the behavioral data and the acquisition of fMRI data was < 1 year.
Non-participation	Data from 4 children were excluded because they dropped out of the study after participating only once for various reasons (for example because the child got braces or the family moved away).
Randomization	Participants were not allocated into experimental groups.

Reporting for specific materials, systems and methods

We require information from authors about some types of materials, experimental systems and methods used in many studies. Here, indicate whether each material, system or method listed is relevant to your study. If you are not sure if a list item applies to your research, read the appropriate section before selecting a response.

Materials & experimental systems

n/a	Involved in the study	
☒	☐	Antibodies
☒	☐	Eukaryotic cell lines
☒	☐	Palaeontology and archaeology
☒	☐	Animals and other organisms
☒	☐	Clinical data
☒	☐	Dual use research of concern
☒	☐	Plants

Methods

n/a	Involved in the study	
☒	☐	ChIP-seq
☒	☐	Flow cytometry
☐	☒	MRI-based neuroimaging

Magnetic resonance imaging

Experimental design

Design type	Block design with 4 seconds trial duration; In each 4 s trial, subjects viewed 8 different images from a single category.
Design specifications	Participants completed two to three runs of the category experiment. Each run is approx. 5 minutes long.
Behavioral performance measures	Participants were instructed to view the images while fixating on a central dot and perform an oddball task during scanning. Oddball task: press a button when an oddball image containing only a texture pattern appears. We report the median and standard deviation of the task performance.

Acquisition

Imaging type(s)	functional, structural
Field strength	3T
Sequence & imaging parameters	MRI data were acquired at the Center for Cognitive Neurobiological Imaging at Stanford University on a 3 Tesla GE Discovery MR750 scanner (GE Medical Systems) using a phase-array 32 channel head coil. Whole brain anatomical scans were collected using quantitative MRI (qMRI, Mezer, A. et al. Quantifying the local tissue volume and composition in individual brains with magnetic resonance imaging. Nat. Med. 19, 1667–1672 (2013)) with a spoiled gradient echo sequence using multiple flip angles (α=4°, 10°, 20°, 30°), TR=14ms and TE=2.4ms. The scan resolution was 0.8x0.8x1.0mm3 (later resampled to 1mm isotropic). For T1-calibration we acquired spin-echo inversion recovery scans with an echo-planar imaging read-out, spectral spatial fat suppression and a slab inversion pulse. These scans were acquired at TR=3s, inplane resolution=2mmx2mm, slice thickness=4mm and 2x acceleration, echo time=minimum full. Functional data were collected using the same scanner and head coil as the structural images. Slices were oriented parallel to the parieto-occipital sulcus. The simultaneous multi-slice, one-shot T2* sensitive gradient echo EPI sequence was acquired with a multiplexing factor of 3 to acquire near whole brain coverage (48 slices), FOV=192mm, TR=1s, TE=30ms, and flip angle=76°. Resolution was 2.4 mm isotropic.
Area of acquisition	Whole brain scans were performed for structural data, near whole brain coverage for functional data.

Diffusion MRI ☐ Used ☒ Not used

Preprocessing

Preprocessing software	Functional data were analyzed using mrVista (https://github.com/vistalab/vistasoft/wiki/mrVista). Motion correction was performed both within and across functional runs. No spatial smoothing and no slice-timing correction were performed. Time courses were transformed into percentage signal change by dividing each timepoint of each voxel's data by the average response across the entire run and then multiplying by 100.
Normalization	Data were not normalized. Functional data from each session were aligned to the individual subject's across years within-subject template. This within-subject template was created using individual T1 brain volumes from the child's multiple timepoints. Each participant's brain anatomical template was generated using the FreeSurfer Longitudinal pipeline (https://surfer.nmr.mgh.harvard.edu/fswiki/LongitudinalProcessing) using FreeSurfer version 6.0.
Normalization template	The data were not normalized to a template. Individual within-subject templates were created and functional data of each participant were aligned to the individual template (see above).
Noise and artifact removal	Motion correction was performed both within and across functional runs.
Volume censoring	n/a

Statistical modeling & inference

Model type and settings	Linear mixed models (LMMs) were used for statistical analyses because (i) the data has a hierarchical structure with sessions being nested within each participant, and (ii) sessions were unevenly distributed across time. Models were fitted using the 'fitlme' function in MATLAB version 2017b (The MathWorks, Inc.). The LMMs were random-intercept models with age as a fixed effect and participant as a random effect (Fig. 1 & 2). Random-slope models were used to predict behavior from distinctiveness with participant as a random effect in analyses shown in Fig. 4.
Effect(s) tested	In analyses related to Figs. 1&2 we tested if category distinctiveness is related to age using the linear mixed models. In analyses related to Fig. 4 we tested if reading and face recognition performance can be predicted using category distinctiveness for words and faces, respectively.

Specify type of analysis: ☐ Whole brain ☒ ROI-based ☐ Both

Anatomical location(s)	Anatomical ROIs (lateral and medial ventral temporal cortex) were individually defined in each participant's native brain space using anatomical landmarks.
Statistic type for inference (See Eklund et al. 2016)	All GLM model parameters and contrasts are computed at the voxel-level; there is no cluster size correction
Correction	FDR for linear mixed models

Models & analysis

n/a	Involved in the study
☒	☐ Functional and/or effective connectivity
☒	☐ Graph analysis
☐	☒ Multivariate modeling or predictive analysis

Multivariate modeling and predictive analysis

We used a multivariate pattern analysis (MVPA) and representational similarity analysis (RSA) to evaluate the representation of category information in ventral temporal cortex over time. We used a distinctiveness metric - within category-similarity minus between-category-similarity to assess the longitudinal development of category information in children's brains. We used also multidimensional scaling (MDS, Fig 3 and supplemental movie) to visualize the internal representation space of children and how it changes across childhood development. Finally, we used a leave-one participant-out-cross validation (LOOCV) approach to test if we can predict behavior (face recognition, reading) from brain data. That is, we computed a LMM that predicts behavior using category distinctiveness on all sessions except for one subject that was left out in each iteration. Then, we used the LMM estimates to compute the predicted behavioral score for each session of the left-out subject. The prediction error was defined as the difference between the predicted and the actual behavioral score. We then repeated this procedure for all subjects.
