# Supplementary material for: Longitudinal development of category representations in ventral temporal cortex predicts word and face recognition
Source: Nat Commun. 2023 Dec 4;14:8010. doi: 10.1038/s41467-023-43146-w (PMC10696026; doi:10.1038/s41467-023-43146-w)
Supplement: Supplementary file 6 — Appendix [file 41467_2023_43146_MOESM6_ESM.docx]

**Appendix**

**Table 1.** Parameters and statistics of LMMs on changes in distinctiveness in lateral VTC ROIs per year. Related to Fig 1. FDR-corrected p-values are reported to adjust for multiple comparisons.

| ROI | Contrast | parameter | β | CI | df | t | *p* | *p FDR* |
| --- | --- | --- | --- | --- | --- | --- | --- | --- |
| Lh lateral VTC | Numbers | intercept | -0.12 | -0.43; 0.19 | 125 | -0.74 | 0.46 |  |
|  |  | tSNR | 0.004 | 0.001;0.007 | 125 | 2.70 | 0.008 |  |
|  |  | age | 0.011 | -0.005;0.026 | 125 | 1.39 | 0.17 | 0.32 |
| n=128 sessions | Words | intercept | 0.08 | -0.22;0.38 | 125 | 0.52 | 0.60 |  |
|  |  | tSNR | 0.002 | -0.001;0.005 | 125 | 1.21 | 0.23 |  |
|  |  | age | 0.026 | 0.011;0.041 | 125 | 3.49 | 0.0007 | 0.0038 |
|  | Limbs | intercept | 0.36 | 0.08;0.64 | 125 | 2.53 | 0.013 |  |
|  |  | tSNR | 0.003 | 0.0006;0.006 | 125 | 2.43 | 0.016 |  |
|  |  | age | -0.006 | -0.020;0.008 | 125 | -0.88 | 0.38 | 0.54 |
|  | Headless bodies | intercept | 0.11 | -0.14;0.36 | 125 | 0.86 | 0.39 |  |
|  |  | tSNR | 0.0017 | -0.0008;0.004 | 125 | 1.35 | 0.18 |  |
|  |  | age | 0.005 | -0.007;0.018 | 125 | 0.89 | 0.38 | 0.54 |
|  | Adult faces | intercept | -0.009 | -0.28;0.26 | 125 | -0.07 | 0.95 |  |
|  |  | tSNR | 0.004 | 0.0012;0.0065 | 125 | 2.89 | 0.005 |  |
|  |  | age | 0.018 | 0.0049;0.031 | 125 | 2.72 | 0.008 | 0.019 |
|  | Child faces | intercept | -0.12 | -0.4;0.16 | 125 | -0.83 | 0.41 |  |
|  |  | tSNR | 0.005 | 0.002;0.007 | 125 | 3.16 | 0.002 |  |
|  |  | age | 0.029 | 0.015;0.043 | 125 | 4.06 | 8.49x10-5 | 0.0017 |
|  | Cars | intercept | -0.14 | -0.41;0.13 | 125 | -1.01 | 0.31 |  |
|  |  | tSNR | 0.002 | -0.0007;0.005 | 125 | 1.51 | 0.13 |  |
|  |  | age | 4.4x10-5 | -0.013;0.014 | 125 | 0.006 | 0.99 | 0.99 |
|  | Instruments | intercept | -0.31 | -0.59; -0.04 | 125 | -2.24 | 0.027 |  |
|  |  | tSNR | 0.006 | 0.003;0.009 | 125 | 4.39 | 2.36x10-5 |  |
|  |  | age | 0.001 | -0.012;0.015 | 125 | 0.17 | 0.86 | 0.93 |
|  | Houses | intercept | -0.40 | -0.73;-0.07 | 125 | -2.42 | 0.017 |  |
|  |  | tSNR | 0.005 | 0.001;0.008 | 125 | 2.72 | 0.008 |  |
|  |  | age | 0.028 | 0.012;0.045 | 125 | 3.45 | 0.0008 | 0.0038 |
|  | Corridors | intercept | 0.17 | -0.10;0.45 | 125 | 1.26 | 0.21 |  |
|  |  | tSNR | 0.003 | 0.0003;0.006 | 125 | 2.18 | 0.03 |  |
|  |  | age | 0.008 | -0.006;0.021 | 125 | 1.12 | 0.27 | 0.44 |
| Rh lateral VTC | Numbers | intercept | -0.28 | -0.55;-0.01 | 125 | -2.08 | 0.0397 |  |
|  |  | tSNR | 0.005 | 0.002;0.007 | 125 | 3.20 | 0.002 |  |
|  |  | age | 0.023 | 0.009;0.037 | 125 | 3.36 | 0.001 | 0.004 |
| n=128 sessions | Words | intercept | 0.28 | 0.014;0.549 | 125 | 2.08 | 0.039 |  |
|  |  | tSNR | -0.001 | -0.004;0.001 | 125 | -0.94 | 0.35 |  |
|  |  | age | 0.009 | -0.004;0.023 | 125 | 1.36 | 0.18 | 0.32 |
|  | Limbs | intercept | 0.31 | 0.015;0.598 | 125 | 2.08 | 0.039 |  |
|  |  | tSNR | 0.005 | 0.002;0.008 | 125 | 3.40 | 0.0009 |  |
|  |  | age | -0.018 | -0.033;-0.004 | 125 | -2.45 | 0.016 | 0.035 |
|  | Headless bodies | intercept | 0.03 | -0.23;0.29 | 125 | 0.23 | 0.82 |  |
|  |  | tSNR | 0.005 | 0.002;0.008 | 125 | 3.66 | 0.0004 |  |
|  |  | age | 0.0017 | -0.012;0.015 | 125 | 0.25 | 0.80 | 0.93 |
|  | Adult faces | intercept | -0.195 | -0.47;0.077 | 125 | -1.42 | 0.16 |  |
|  |  | tSNR | 0.008 | 0.005;0.011 | 125 | 5.35 | 4.02x10-7 |  |
|  |  | age | 0.0199 | 0.006;0.034 | 125 | 2.85 | 0.005 | 0.015 |
|  | Child faces | intercept | -0.09 | -0.34;0.16 | 125 | -0.70 | 0.48 |  |
|  |  | tSNR | 0.006 | 0.004;0.009 | 125 | 4.86 | 3.4x10-6 |  |
|  |  | age | 0.025 | 0.012;0.038 | 125 | 3.84 | 0.0002 | 0.002 |
|  | Cars | intercept | -0.05 | -0.35;0.24 | 125 | -0.35 | 0.73 |  |
|  |  | tSNR | 0.001 | -0.002;0.004 | 125 | 0.77 | 0.44 |  |
|  |  | age | -0.001 | -0.016;0.014 | 125 | -0.15 | 0.88 | 0.93 |
|  | Instruments | intercept | -0.04 | -0.27;0.18 | 125 | -0.39 | 0.697 |  |
|  |  | tSNR | 0.003 | 0.0003;0.005 | 125 | 2.23 | 0.027 |  |
|  |  | age | 0.0019 | -0.009;0.013 | 125 | 0.34 | 0.74 | 0.92 |
|  | Houses | intercept | -0.31 | -0.65;0.03 | 125 | -1.79 | 0.08 |  |
|  |  | tSNR | 0.006 | 0.002;0.009 | 125 | 3.22 | 0.002 |  |
|  |  | age | 0.026 | 0.009;0.043 | 125 | 2.98 | 0.003 | 0.011 |
|  | Corridors | intercept | 0.21 | -0.027;0.454 | 125 | 1.76 | 0.08 |  |
|  |  | tSNR | 0.005 | 0.002;0.007 | 125 | 3.54 | 0.0006 |  |
|  |  | age | 0.003 | -0.009;0.015 | 125 | 0.52 | 0.61 | 0.81 |

**Table 2.** Parameters and statistics of LMMs on changes in distinctiveness in medial VTC ROIs per year. Related to Fig 1. FDR-corrected p-values are reported to adjust for multiple comparisons.

| ROI | Contrast | parameter | β | CI | df | t | *p* | *p FDR* |
| --- | --- | --- | --- | --- | --- | --- | --- | --- |
| Lh medial VTC | Numbers | intercept | -0.196 | -0.45,0.06 | 125 | -1.5 | 0.14 |  |
|  |  | tSNR | 0.007 | 0.004;0.01 | 125 | 4.28 | 3.62x10-5 |  |
|  |  | age | 0.0005 | -0.011;0.012 | 125 | 0.09 | 0.93 | 0.98 |
| n=128 sessions | Words | intercept | 0.27 | -0.05;0.59 | 125 | 1.65 | 0.10 |  |
|  |  | tSNR | -0.0003 | -0.004;0.004 | 125 | -0.15 | 0.88 |  |
|  |  | age | 0.002 | -0.012;0.016 | 125 | 0.29 | 0.77 | 0.98 |
|  | Limbs | intercept | -0.016 | -0.32;0.29 | 125 | -0.10 | 0.92 |  |
|  |  | tSNR | 0.003 | -0.0002;0.007 | 125 | 1.89 | 0.06 |  |
|  |  | age | -0.0019 | -0.015;0.011 | 125 | -0.29 | 0.78 | 0.98 |
|  | Headless bodies | intercept | -0.12 | -0.38;0.14 | 125 | -0.91 | 0.36 |  |
|  |  | tSNR | 0.005 | 0.002;0.008 | 125 | 3.34 | 0.001 |  |
|  |  | age | 0.0007 | -0.01;0.01 | 125 | 0.13 | 0.9 | 0.98 |
|  | Adult faces | intercept | -0.013 | -0.28;0.26 | 125 | -0.098 | 0.92 |  |
|  |  | tSNR | 0.005 | 0.002;0.008 | 125 | 2.95 | 0.004 |  |
|  |  | age | 0.0186 | 0.007;0.03 | 125 | 3.048 | 0.0028 | 0.028 |
|  | Child faces | intercept | -0.098 | -0.393;0.197 | 125 | -0.66 | 0.51 |  |
|  |  | tSNR | 0.006 | 0.003;0.009 | 125 | 3.59 | 0.0005 |  |
|  |  | age | 0.0187 | 0.005;0.03 | 125 | 2.76 | 0.0067 | 0.034 |
|  | Cars | intercept | -0.25 | -0.53;0.03 | 125 | -1.76 | 0.08 |  |
|  |  | tSNR | 0.005 | 0.002;0.008 | 125 | 2.88 | 0.005 |  |
|  |  | age | -0.004 | -0.016;0.009 | 125 | -0.60 | 0.55 | 0.98 |
|  | Instruments | intercept | -0.195 | -0.41;0.023 | 125 | -1.77 | 0.079 |  |
|  |  | tSNR | 0.005 | 0.0025;0.0078 | 125 | 3.82 | 0.0002 |  |
|  |  | age | 0.001 | -0.008;0.0105 | 125 | 0.21 | 0.84 | 0.98 |
|  | Houses | intercept | -0.61 | -0.98;-0.24 | 125 | -3.27 | 0.001 |  |
|  |  | tSNR | 0.013 | 0.009;0.018 | 125 | 6.08 | 1.38x10-8 |  |
|  |  | age | 0.023 | 0.006;0.039 | 125 | 2.76 | 0.0067 | 0.034 |
|  | Corridors | intercept | 0.13 | -0.12;0.39 | 125 | 1.02 | 0.31 |  |
|  |  | tSNR | 0.01 | 0.007;0.013 | 125 | 6.57 | 1.19x10-9 |  |
|  |  | age | -0.002 | -0.013;0.009 | 125 | -0.38 | 0.70 | 0.98 |
| Rh medial VTC | Numbers | intercept | -0.433 | -0.72;-0.14 | 125 | -2.97 | 0.0035 |  |
|  |  | tSNR | 0.008 | 0.004;0.012 | 125 | 4.05 | 8.91x10-5 |  |
|  |  | age | 0.011 | -0.0006;0.023 | 125 | 1.89 | 0.06 | 0.21 |
| n=128 sessions | Words | intercept | 0.45 | 0.15;0.76 | 125 | 2.95 | 0.0038 |  |
|  |  | tSNR | -0.0058 | -0.0099;-0.002 | 125 | -2.80 | 0.0059 |  |
|  |  | age | -0.0003 | -0.013;0.012 | 125 | -0.05 | 0.96 | 0.98 |
|  | Limbs | intercept | -0.29 | -0.57;-0.008 | 125 | -2.04 | 0.04 |  |
|  |  | tSNR | 0.007 | 0.0029;0.010 | 125 | 3.5 | 0.0006 |  |
|  |  | age | 0.006 | -0.005;0.018 | 125 | 1.06 | 0.29 | 0.58 |
|  | Headless bodies | intercept | -0.22 | -0.54;0.10 | 125 | -1.35 | 0.18 |  |
|  |  | tSNR | 0.006 | 0.002;0.010 | 125 | 2.80 | 0.006 |  |
|  |  | age | 0.008 | -0.006;0.022 | 125 | 1.19 | 0.24 | 0.52 |
|  | Adult faces | intercept | -0.036 | -0.43;0.36 | 125 | -0.18 | 0.86 |  |
|  |  | tSNR | 0.006 | 0.0009;0.011 | 125 | 2.31 | 0.02 |  |
|  |  | age | 0.013 | -0.004;0.030 | 125 | 1.56 | 0.12 | 0.30 |
|  | Child faces | intercept | 0.018 | -0.33;0.36 | 125 | 0.10 | 0.92 |  |
|  |  | tSNR | 0.0045 | -5.4x10-5;0.01 | 125 | 1.96 | 0.053 |  |
|  |  | age | 0.014 | -0.0006;0.029 | 125 | 1.89 | 0.061 | 0.21 |
|  | Cars | intercept | -0.036 | -0.39;0.32 | 125 | -0.20 | 0.84 |  |
|  |  | tSNR | 0.001 | -0.004;0.006 | 125 | 0.42 | 0.68 |  |
|  |  | age | 0.0002 | -0.015;0.015 | 125 | 0.03 | 0.98 | 0.98 |
|  | Instruments | intercept | -0.18 | -0.45;0.10 | 125 | -1.26 | 0.21 |  |
|  |  | tSNR | 0.006 | 0.002;0.009 | 125 | 3.09 | 0.002 |  |
|  |  | age | -0.0009 | -0.01;0.01 | 125 | -0.15 | 0.88 | 0.98 |
|  | Houses | intercept | -0.45 | -0.86;-0.03 | 125 | -2.14 | 0.035 |  |
|  |  | tSNR | 0.011 | 0.006;0.016 | 125 | 4.06 | 8.5x10-5 |  |
|  |  | age | 0.032 | 0.015;0.050 | 125 | 3.61 | 0.0004 | 0.009 |
|  | Corridors | intercept | 0.14 | -0.13;0.42 | 125 | 1.04 | 0.3 |  |
|  |  | tSNR | 0.009 | 0.005;0.012 | 125 | 4.96 | 2.2x10-6 |  |
|  |  | age | 0.010 | -0.0015;0.022 | 125 | 1.72 | 0.087 | 0.25 |

**Table 3.** Parameters and statistics of LMMs on changes in distinctiveness in the union of selective voxels in lateral VTC ROIs per year. Related to Fig 2. FDR-corrected p-values are reported to adjust for multiple comparisons.

| ROI | Contrast | parameter | β | CI | df | t | *p* | *p FDR* |
| --- | --- | --- | --- | --- | --- | --- | --- | --- |
| Lh lateral VTC | Numbers | intercept | 0.0003 | -0.34;0.34 | 125 | 0.002 | 0.998 |  |
|  |  | tSNR | 0.004 | 0.0007;0.008 | 125 | 2.397 | 0.018 |  |
|  |  | age | 0.016 | 0.0002;0.032 | 125 | 1.998 | 0.048 | 0.21 |
| n=128 sessions | Words | intercept | 0.35 | 0.082;0.624 | 125 | 2.57 | 0.011 |  |
|  |  | tSNR | 0.0005 | -0.002;0.003 | 125 | 0.38 | 0.70 |  |
|  |  | age | 0.026 | 0.013;0.039 | 125 | 3.99 | 0.0001 | 0.002 |
|  | Limbs | intercept | 0.55 | 0.278;0.82 | 125 | 4.00 | 0.0001 |  |
|  |  | tSNR | 0.002 | -0.0007;0.005 | 125 | 1.47 | 0.15 |  |
|  |  | age | 0.0003 | -0.01;0.01 | 125 | 0.054 | 0.96 | 0.96 |
|  | Headless bodies | intercept | 0.25 | -0.037;0.53 | 125 | 1.72 | 0.09 |  |
|  |  | tSNR | 0.002 | -0.001;0.004 | 125 | 1.14 | 0.26 |  |
|  |  | age | 0.009 | -0.005;0.022 | 125 | 1.31 | 0.19 | 0.50 |
|  | Adult faces | intercept | 0.078 | -0.21;0.36 | 125 | 0.54 | 0.59 |  |
|  |  | tSNR | 0.004 | 0.001;0.007 | 125 | 2.96 | 0.004 |  |
|  |  | age | 0.026 | 0.013;0.039 | 125 | 3.88 | 0.0002 | 0.002 |
|  | Child faces | intercept | 0.11 | -0.22;0.44 | 125 | 0.67 | 0.50 |  |
|  |  | tSNR | 0.003 | 0.0001;0.007 | 125 | 2.07 | 0.04 |  |
|  |  | age | 0.034 | 0.019;0.05 | 125 | 4.39 | 2.36x10-5 | 0.0009 |
|  | Cars | intercept | -0.009 | -0.31;0.29 | 125 | -0.06 | 0.95 |  |
|  |  | tSNR | 0.001 | -0.002;0.004 | 125 | 0.85 | 0.4 |  |
|  |  | age | -0.0004 | -0.014;0.013 | 125 | -0.062 | 0.95 | 0.96 |
|  | Instruments | intercept | -0.25 | -0.60;0.09 | 125 | -1.46 | 0.15 |  |
|  |  | tSNR | 0.006 | 0.003;0.01 | 125 | 3.62 | 0.0004 |  |
|  |  | age | 0.005 | -0.011;0.021 | 125 | 0.64 | 0.52 | 0.79 |
|  | Houses | intercept | -0.30 | -0.72;0.11 | 125 | -1.45 | 0.15 |  |
|  |  | tSNR | 0.003 | -0.0009;0.007 | 125 | 1.54 | 0.13 |  |
|  |  | age | 0.036 | 0.017;0.06 | 125 | 3.69 | 0.0003 | 0.003 |
|  | Corridors | intercept | 0.35 | 0.034;0.67 | 125 | 2.19 | 0.03 |  |
|  |  | tSNR | 0.001 | -0.002;0.004 | 125 | 0.85 | 0.4 |  |
|  |  | age | 0.01 | -0.005;0.025 | 125 | 1.32 | 0.19 | 0.50 |
| Rh lateral VTC | Numbers | intercept | 0.049 | -0.33;0.43 | 125 | 0.26 | 0.797 |  |
|  |  | tSNR | 0.0018 | -0.002;0.006 | 125 | 0.90 | 0.37 |  |
|  |  | age | 0.027 | 0.0098;0.045 | 125 | 3.08 | 0.0025 | 0.017 |
| n=128 sessions | Words | intercept | 0.62 | 0.28;0.96 | 125 | 3.58 | 0.0005 |  |
|  |  | tSNR | -0.003 | -0.006;0.0005 | 125 | -1.68 | 0.1 |  |
|  |  | age | 0.005 | -0.01;0.02 | 125 | 0.65 | 0.52 | 0.79 |
|  | Limbs | intercept | 0.53 | 0.21;0.86 | 125 | 3.25 | 0.001 |  |
|  |  | tSNR | 0.004 | 0.0002;0.007 | 125 | 2.11 | 0.037 |  |
|  |  | age | -0.013 | -0.028;0.002 | 125 | -1.67 | 0.098 | 0.33 |
|  | Headless bodies | intercept | 0.12 | -0.19;0.43 | 125 | 0.75 | 0.46 |  |
|  |  | tSNR | 0.006 | 0.002;0.009 | 125 | 3.46 | 0.0007 |  |
|  |  | age | 0.007 | -0.008;0.021 | 125 | 0.89 | 0.37 | 0.65 |
|  | Adult faces | intercept | 0.33 | 0.03;0.62 | 125 | 2.20 | 0.03 |  |
|  |  | tSNR | 0.004 | 0.0007;0.007 | 125 | 2.44 | 0.016 |  |
|  |  | age | 0.02 | 0.007;0.034 | 125 | 2.95 | 0.004 | 0.02 |
|  | Child faces | intercept | 0.51 | 0.24;0.77 | 125 | 3.78 | 0.0002 |  |
|  |  | tSNR | 0.0028 | -1.4x10-6;0.006 | 125 | 1.98 | 0.05 |  |
|  |  | age | 0.017 | 0.0048;0.03 | 125 | 2.75 | 0.007 | 0.03 |
|  | Cars | intercept | -0.05 | -0.43;0.33 | 125 | -0.24 | 0.81 |  |
|  |  | tSNR | 0.0018 | -0.002;0.006 | 125 | 0.88 | 0.38 |  |
|  |  | age | 0.0009 | -0.016;0.018 | 125 | 0.1 | 0.92 | 0.96 |
|  | Instruments | intercept | 0.13 | -0.19;0.45 | 125 | 0.80 | 0.42 |  |
|  |  | tSNR | 0.0027 | -0.0007;0.006 | 125 | 1.56 | 0.12 |  |
|  |  | age | -0.004 | -0.019;0.011 | 125 | -0.49 | 0.63 | 0.79 |
|  | Houses | intercept | -0.05 | -0.47;0.36 | 125 | -0.26 | 0.80 |  |
|  |  | tSNR | 0.003 | -0.0007;0.008 | 125 | 1.62 | 0.11 |  |
|  |  | age | 0.034 | 0.015;0.054 | 125 | 3.49 | 0.0007 | 0.005 |
|  | Corridors | intercept | 0.47 | 0.16;0.78 | 125 | 3.03 | 0.003 |  |
|  |  | tSNR | 0.002 | -0.001;0.005 | 125 | 1.33 | 0.19 |  |
|  |  | age | 0.009 | -0.005;0.02 | 125 | 1.23 | 0.22 | 0.52 |

**Table 4.** Parameters and statistics of LMMs on changes in distinctiveness in the non-selective voxels in lateral VTC ROIs per year. Related to Fig 2. FDR-corrected p-values are reported to adjust for multiple comparisons.

| ROI | Contrast | parameter | β | CI | df | t | *p* | *p FDR* |
| --- | --- | --- | --- | --- | --- | --- | --- | --- |
| Lh lateral VTC | Numbers | intercept | -0.05 | -0.26;0.16 | 125 | -0.44 | 0.66 |  |
|  |  | tSNR | 0.0009 | -0.001;0.003 | 125 | 0.86 | 0.39 |  |
|  |  | age | 0.005 | -0.006;0.017 | 125 | 0.96 | 0.34 | 0.64 |
| n=128 sessions | Words | intercept | 0.14 | -0.06;0.34 | 125 | 1.37 | 0.17 |  |
|  |  | tSNR | 0.0004 | -0.002;0.002 | 125 | 0.35 | 0.72 |  |
|  |  | age | 0.0005 | -0.01;0.01 | 125 | 0.09 | 0.93 | 0.96 |
|  | Limbs | intercept | 0.228 | -0.012;0.47 | 125 | 1.88 | 0.06 |  |
|  |  | tSNR | -0.0002 | -0.003;0.002 | 125 | -0.197 | 0.84 |  |
|  |  | age | -0.004 | -0.016;0.009 | 125 | -0.56 | 0.57 | 0.79 |
|  | Headless bodies | intercept | 0.066 | -0.12;0.25 | 125 | 0.69 | 0.49 |  |
|  |  | tSNR | -0.0002 | -0.002;0.002 | 125 | -0.18 | 0.86 |  |
|  |  | age | 0.004 | -0.006;0.014 | 125 | 0.79 | 0.43 | 0.72 |
|  | Adult faces | intercept | 0.2 | -0.04;0.44 | 125 | 1.64 | 0.10 |  |
|  |  | tSNR | 0.0003 | -0.002;0.003 | 125 | 0.22 | 0.83 |  |
|  |  | age | -0.0009 | -0.014;0.012 | 125 | -0.14 | 0.89 | 0.96 |
|  | Child faces | intercept | 0.14 | -0.12;0.39 | 125 | 1.07 | 0.29 |  |
|  |  | tSNR | 4.98x10-5 | -0.002;0.003 | 125 | 0.04 | 0.97 |  |
|  |  | age | 0.007 | -0.007;0.02 | 125 | 0.97 | 0.33 | 0.64 |
|  | Cars | intercept | -0.12 | -0.34;0.11 | 125 | -1.03 | 0.31 |  |
|  |  | tSNR | 0.0015 | -0.0008;0.004 | 125 | 1.31 | 0.19 |  |
|  |  | age | -0.002 | -0.01;0.01 | 125 | -0.399 | 0.69 | 0.81 |
|  | Instruments | intercept | -0.096 | -0.29;0.100 | 125 | -0.97 | 0.33 |  |
|  |  | tSNR | 0.002 | 0.0004;0.0044 | 125 | 2.43 | 0.017 |  |
|  |  | age | -0.003 | -0.013;0.008 | 125 | -0.51 | 0.61 | 0.79 |
|  | Houses | intercept | -0.11 | -0.35;0.12 | 125 | -0.95 | 0.34 |  |
|  |  | tSNR | 0.001 | -0.001;0.004 | 125 | 1.08 | 0.28 |  |
|  |  | age | 0.008 | -0.004;0.02 | 125 | 1.31 | 0.19 | 0.50 |
|  | Corridors | intercept | 0.24 | 0.018;0.469 | 125 | 2.13 | 0.035 |  |
|  |  | tSNR | 0.0005 | -0.002;0.003 | 125 | 0.442 | 0.66 |  |
|  |  | age | -0.003 | -0.015;0.009 | 125 | -0.48 | 0.63 | 0.79 |
| Rh lateral VTC | Numbers | intercept | -0.17 | -0.34;-0.005 | 125 | -2.04 | 0.04 |  |
|  |  | tSNR | 0.002 | 0.0007;0.004 | 125 | 2.75 | 0.007 |  |
|  |  | age | 0.009 | -0.0004;0.019 | 125 | 1.9 | 0.06 | 0.24 |
| n=128 sessions | Words | intercept | 0.14 | -0.05;0.32 | 125 | 1.47 | 0.14 |  |
|  |  | tSNR | -0.001 | -0.003;0.0006 | 125 | -1.37 | 0.17 |  |
|  |  | age | 0.006 | -0.004;0.016 | 125 | 1.12 | 0.27 | 0.59 |
|  | Limbs | intercept | 0.07 | -0.13;0.27 | 125 | 0.7 | 0.49 |  |
|  |  | tSNR | 0.002 | 3.4x10-5;0.004 | 125 | 2.01 | 0.046 |  |
|  |  | age | -0.005 | -0.016;0.006 | 125 | -0.89 | 0.37 | 0.65 |
|  | Headless bodies | intercept | 0.05 | -0.12;0.22 | 125 | 0.60 | 0.55 |  |
|  |  | tSNR | 0.001 | -0.0005;0.003 | 125 | 1.36 | 0.18 |  |
|  |  | age | 0.002 | -0.007;0.01 | 125 | 0.42 | 0.68 | 0.81 |
|  | Adult faces | intercept | -0.016 | -0.23;0.20 | 125 | -0.14 | 0.89 |  |
|  |  | tSNR | 0.003 | 0.001;0.006 | 125 | 3.02 | 0.003 |  |
|  |  | age | 0.004 | -0.009;0.016 | 125 | 0.57 | 0.57 | 0.79 |
|  | Child faces | intercept | 0.14 | -0.07;0.36 | 125 | 1.3 | 0.195 |  |
|  |  | tSNR | 0.0009 | -0.001;0.003 | 125 | 0.85 | 0.4 |  |
|  |  | age | 0.006 | -0.006;0.018 | 125 | 0.98 | 0.33 | 0.64 |
|  | Cars | intercept | -0.01 | -0.21;0.18 | 125 | -0.11 | 0.91 |  |
|  |  | tSNR | 0.0002 | -0.002;0.002 | 125 | 0.16 | 0.87 |  |
|  |  | age | -0.003 | -0.014;0.008 | 125 | -0.52 | 0.60 | 0.79 |
|  | Instruments | intercept | 0.037 | -0.14;0.21 | 125 | 0.42 | 0.67 |  |
|  |  | tSNR | 0.0004 | -0.001;0.002 | 125 | 0.41 | 0.68 |  |
|  |  | age | 0.0002 | -0.01;0.01 | 125 | 0.049 | 0.96 | 0.96 |
|  | Houses | intercept | -0.13 | -0.35;0.09 | 125 | -1.16 | 0.25 |  |
|  |  | tSNR | 0.003 | 0.0003;0.005 | 125 | 2.23 | 0.027 |  |
|  |  | age | 0.008 | -0.004;0.02 | 125 | 1.28 | 0.20 | 0.50 |
|  | Corridors | intercept | 0.23 | 0.07;0.4 | 125 | 2.78 | 0.006 |  |
|  |  | tSNR | 0.002 | 0.0007;0.004 | 125 | 2.79 | 0.006 |  |
|  |  | age | -0.008 | -0.018;0.0009 | 125 | -1.79 | 0.08 | 0.28 |

**Table 5.** Parameters and statistics of LMMs on changes in distinctiveness in the union of selective voxels in medial VTC ROIs per year. Related to Fig 2. FDR-corrected p-values are reported to adjust for multiple comparisons.

| ROI | Contrast | parameter | β | CI | df | t | *p* | *p FDR* |
| --- | --- | --- | --- | --- | --- | --- | --- | --- |
| Lh medial VTC | Numbers | intercept | 0.048 | -0.30;0.396 | 125 | 0.27 | 0.78 |  |
|  |  | tSNR | 0.005 | 0.0006;0.009 | 125 | 2.26 | 0.025 |  |
|  |  | age | 0.0066 | -0.009;0.023 | 125 | 0.81 | 0.42 | 0.84 |
| n=128 sessions | Words | intercept | 0.67 | 0.27;1.07 | 125 | 3.33 | 0.001 |  |
|  |  | tSNR | -0.004 | -0.009;0.001 | 125 | -1.52 | 0.13 |  |
|  |  | age | 0.001 | -0.018;0.02 | 125 | 0.11 | 0.91 | 0.98 |
|  | Limbs | intercept | 0.18 | -0.22;0.58 | 125 | 0.91 | 0.37 |  |
|  |  | tSNR | 0.002 | -0.003;0.007 | 125 | 0.88 | 0.38 |  |
|  |  | age | -0.0004 | -0.019;0.018 | 125 | -0.044 | 0.96 | 0.99 |
|  | Headless bodies | intercept | 0.12 | -0.24;0.49 | 125 | 0.68 | 0.496 |  |
|  |  | tSNR | 0.004 | -9.x10-5;0.009 | 125 | 1.94 | 0.055 |  |
|  |  | age | -0.007 | -0.023;0.01 | 125 | -0.77 | 0.44 | 0.84 |
|  | Adult faces | intercept | 0.28 | -0.064;0.64 | 125 | 1.61 | 0.11 |  |
|  |  | tSNR | 0.0028 | -0.001;0.007 | 125 | 1.35 | 0.18 |  |
|  |  | age | 0.015 | -0.0014;0.032 | 125 | 1.81 | 0.07 | 0.46 |
|  | Child faces | intercept | 0.26 | -0.11;0.63 | 125 | 1.40 | 0.16 |  |
|  |  | tSNR | 0.003 | -0.0007;0.008 | 125 | 1.65 | 0.10 |  |
|  |  | age | 0.0155 | -0.002;0.033 | 125 | 1.74 | 0.085 | 0.46 |
|  | Cars | intercept | -0.15 | -0.53;0.23 | 125 | -0.77 | 0.44 |  |
|  |  | tSNR | 0.006 | 0.002;0.011 | 125 | 2.67 | 0.008 |  |
|  |  | age | -0.015 | -0.03;0.002 | 125 | -1.70 | 0.09 | 0.46 |
|  | Instruments | intercept | -0.037 | -0.33;0.26 | 125 | -0.25 | 0.80 |  |
|  |  | tSNR | 0.005 | 0.0009;0.008 | 125 | 2.47 | 0.015 |  |
|  |  | age | -0.0035 | -0.017;0.01 | 125 | -0.51 | 0.61 | 0.94 |
|  | Houses | intercept | -0.09 | -0.49;0.30 | 125 | -0.46 | 0.64 |  |
|  |  | tSNR | 0.01 | 0.006;0.015 | 125 | 4.33 | 2.98x10-5 |  |
|  |  | age | 0.007 | -0.011;0.026 | 125 | 0.77 | 0.44 | 0.84 |
|  | Corridors | intercept | 0.66 | 0.40;0.92 | 125 | 5.07 | 1.39x10-6 |  |
|  |  | tSNR | 0.005 | 0.0019;0.008 | 125 | 3.17 | 0.0019 |  |
|  |  | age | -0.003 | -0.015;0.009 | 125 | -0.56 | 0.57 | 0.94 |
| Rh medial VTC | Numbers | intercept | -0.349 | -0.72;0.03 | 125 | -1.845 | 0.067 |  |
|  |  | tSNR | 0.008 | 0.003;0.01 | 125 | 3.36 | 0.001 |  |
|  |  | age | 0.014 | -0.003;0.03 | 125 | 1.64 | 0.10 | 0.46 |
| n=128 sessions | Words | intercept | 0.55 | 0.12;0.97 | 125 | 2.53 | 0.013 |  |
|  |  | tSNR | -0.007 | -0.012;-0.0009 | 125 | -2.29 | 0.024 |  |
|  |  | age | 0.0016 | -0.018;0.02 | 125 | 0.16 | 0.87 | 0.98 |
|  | Limbs | intercept | -0.27 | -0.63;0.09 | 125 | -1.48 | 0.14 |  |
|  |  | tSNR | 0.007 | 0.002;0.012 | 125 | 2.93 | 0.004 |  |
|  |  | age | 0.013 | -0.003;0.029 | 125 | 1.59 | 0.11 | 0.46 |
|  | Headless bodies | intercept | -0.16 | -0.55;0.24 | 125 | -0.78 | 0.43 |  |
|  |  | tSNR | 0.007 | 0.002;0.012 | 125 | 2.81 | 0.006 |  |
|  |  | age | 0.0028 | -0.015;0.021 | 125 | 0.311 | 0.76 | 0.94 |
|  | Adult faces | intercept | 0.077 | -0.341;0.495 | 125 | 0.36 | 0.72 |  |
|  |  | tSNR | 0.005 | -0.0003;0.011 | 125 | 1.86 | 0.065 |  |
|  |  | age | 0.014 | -0.005;0.033 | 125 | 1.45 | 0.15 | 0.52 |
|  | Child faces | intercept | 0.047 | -0.34;0.43 | 125 | 0.24 | 0.81 |  |
|  |  | tSNR | 0.005 | 0.0003;0.01 | 125 | 2.12 | 0.036 |  |
|  |  | age | 0.013 | -0.005;0.03 | 125 | 1.43 | 0.156 | 0.52 |
|  | Cars | intercept | -0.19 | -0.611;0.23 | 125 | -0.91 | 0.37 |  |
|  |  | tSNR | 0.003 | -0.002;0.009 | 125 | 1.21 | 0.23 |  |
|  |  | age | 0.004 | -0.015;0.023 | 125 | 0.41 | 0.69 | 0.94 |
|  | Instruments | intercept | 0.24 | -0.09;0.58 | 125 | 1.43 | 0.15 |  |
|  |  | tSNR | -0.0004 | -0.005;0.004 | 125 | -0.19 | 0.85 |  |
|  |  | age | -0.002 | -0.017;0.013 | 125 | -0.28 | 0.78 | 0.94 |
|  | Houses | intercept | -0.26 | -0.66;0.15 | 125 | -1.26 | 0.21 |  |
|  |  | tSNR | 0.009 | 0.004;0.015 | 125 | 3.38 | 0.00097 |  |
|  |  | age | 0.028 | 0.0097;0.046 | 125 | 3.03 | 0.003 | 0.12 |
|  | Corridors | intercept | 0.599 | 0.296;0.902 | 125 | 3.91 | 0.0001 |  |
|  |  | tSNR | 0.003 | -0.0008;0.007 | 125 | 1.58 | 0.12 |  |
|  |  | age | 0.0039 | -0.01;0.018 | 125 | 0.56 | 0.58 | 0.94 |

**Table 6.** Parameters and statistics of LMMs on changes in distinctiveness in the non-selective voxels in medial VTC ROIs per year. Related to Fig 2. FDR-corrected p-values are reported to adjust for multiple comparisons.

| ROI | Contrast | parameter | β | CI | df | t | *p* | *p FDR* |
| --- | --- | --- | --- | --- | --- | --- | --- | --- |
| Lh medial VTC | Numbers | intercept | -0.14 | -0.34;0.055 | 125 | -1.43 | 0.156 |  |
|  |  | tSNR | 0.004 | 0.002;0.007 | 125 | 3.59 | 0.0005 |  |
|  |  | age | -0.0004 | -0.009;0.008 | 125 | -0.08 | 0.93 | 0.98 |
| n=128 sessions | Words | intercept | 0.159 | -0.056;0.374 | 125 | 1.46 | 0.146 |  |
|  |  | tSNR | -0.0001 | -0.003;0.002 | 125 | -0.099 | 0.92 |  |
|  |  | age | -0.0029 | -0.013;0.007 | 125 | -0.599 | 0.55 | 0.94 |
|  | Limbs | intercept | -0.11 | -0.32;0.11 | 125 | -0.98 | 0.33 |  |
|  |  | tSNR | 0.003 | 0.0004;0.006 | 125 | 2.31 | 0.022 |  |
|  |  | age | 0.0005 | -0.009;0.010 | 125 | 0.11 | 0.91 | 0.98 |
|  | Headless bodies | intercept | -0.06 | -0.24;0.12 | 125 | -0.67 | 0.50 |  |
|  |  | tSNR | 0.002 | 0.0003;0.005 | 125 | 2.24 | 0.027 |  |
|  |  | age | 0.0012 | -0.007;0.009 | 125 | 0.29 | 0.77 | 0.94 |
|  | Adult faces | intercept | -0.099 | -0.32;0.12 | 125 | -0.88 | 0.38 |  |
|  |  | tSNR | 0.0037 | 0.001;0.006 | 125 | 2.88 | 0.0046 |  |
|  |  | age | 0.012 | 0.002;0.022 | 125 | 2.41 | 0.018 | 0.35 |
|  | Child faces | intercept | -0.19 | -0.46;0.08 | 125 | -1.39 | 0.17 |  |
|  |  | tSNR | 0.005 | 0.002;0.008 | 125 | 3.14 | 0.002 |  |
|  |  | age | 0.0128 | 0.0005;0.025 | 125 | 2.06 | 0.041 | 0.41 |
|  | Cars | intercept | -0.14 | -0.38;0.11 | 125 | -1.09 | 0.28 |  |
|  |  | tSNR | 0.003 | -3.2x10-5;0.006 | 125 | 1.96 | 0.053 |  |
|  |  | age | -0.006 | -0.017;0.005 | 125 | -1.05 | 0.29 | 0.69 |
|  | Instruments | intercept | -0.06 | -0.26;0.13 | 125 | -0.65 | 0.51 |  |
|  |  | tSNR | 0.002 | -0.0004;0.004 | 125 | 1.63 | 0.11 |  |
|  |  | age | 0.0008 | -0.008;0.009 | 125 | 0.18 | 0.86 | 0.98 |
|  | Houses | intercept | -0.30 | -0.601;-0.003 | 125 | -1.999 | 0.048 |  |
|  |  | tSNR | 0.005 | 0.0017;0.009 | 125 | 2.95 | 0.0038 |  |
|  |  | age | 0.007 | -0.006;0.021 | 125 | 1.1 | 0.29 | 0.69 |
|  | Corridors | intercept | 0.012 | -0.256;0.28 | 125 | 0.09 | 0.93 |  |
|  |  | tSNR | 0.007 | 0.004;0.01 | 125 | 4.5 | 1.6x10-5 |  |
|  |  | age | -0.01 | -0.022;0.002 | 125 | -1.67 | 0.098 | 0.46 |
| Rh medial VTC | Numbers | intercept | -0.39 | -0.63;-0.15 | 125 | -3.19 | 0.002 |  |
|  |  | tSNR | 0.006 | 0.0027;0.0092 | 125 | 3.63 | 0.0004 |  |
|  |  | age | 0.011 | 0.0009;0.021 | 125 | 2.16 | 0.033 | 0.41 |
| n=128 sessions | Words | intercept | 0.29 | 0.05;0.53 | 125 | 2.43 | 0.017 |  |
|  |  | tSNR | -0.004 | -0.007;-0.0004 | 125 | -2.21 | 0.029 |  |
|  |  | age | -0.0023 | -0.012;0.008 | 125 | -0.45 | 0.66 | 0.94 |
|  | Limbs | intercept | -0.18 | -0.42;0.053 | 125 | -1.53 | 0.13 |  |
|  |  | tSNR | 0.0045 | 0.0013;0.008 | 125 | 2.76 | 0.007 |  |
|  |  | age | 8.2x10-5 | -0.0099;0.010 | 125 | 0.016 | 0.99 | 0.99 |
|  | Headless bodies | intercept | 0.019 | -0.21;0.247 | 125 | 0.16 | 0.87 |  |
|  |  | tSNR | 0.0006 | -0.0025;0.0036 | 125 | 0.375 | 0.71 |  |
|  |  | age | 0.004 | -0.005;0.014 | 125 | 0.86 | 0.39 | 0.84 |
|  | Adult faces | intercept | -0.04 | -0.33;0.25 | 125 | -0.28 | 0.78 |  |
|  |  | tSNR | 0.0039 | 0.0001;0.008 | 125 | 2.06 | 0.042 |  |
|  |  | age | 0.0067 | -0.0059;0.019 | 125 | 1.05 | 0.29 | 0.69 |
|  | Child faces | intercept | 0.044 | -0.25;0.34 | 125 | 0.299 | 0.77 |  |
|  |  | tSNR | 0.0017 | -0.002;0.006 | 125 | 0.86 | 0.39 |  |
|  |  | age | 0.009 | -0.004;0.021 | 125 | 1.37 | 0.17 | 0.53 |
|  | Cars | intercept | -0.029 | -0.30;0.25 | 125 | -0.21 | 0.84 |  |
|  |  | tSNR | -0.0001 | -0.0038;0.0036 | 125 | -0.06 | 0.96 |  |
|  |  | age | 0.0019 | -0.0097;0.013 | 125 | 0.32 | 0.75 | 0.94 |
|  | Instruments | intercept | -0.21 | -0.43;0.002 | 125 | -1.96 | 0.052 |  |
|  |  | tSNR | 0.005 | 0.002;0.008 | 125 | 3.42 | 0.0008 |  |
|  |  | age | -0.002 | -0.011;0.007 | 125 | -0.46 | 0.65 | 0.94 |
|  | Houses | intercept | -0.27 | -0.56;0.02 | 125 | -1.84 | 0.068 |  |
|  |  | tSNR | 0.005 | 0.001;0.009 | 125 | 2.66 | 0.009 |  |
|  |  | age | 0.0072 | -0.005;0.0197 | 125 | 1.15 | 0.25 | 0.69 |
|  | Corridors | intercept | 0.195 | -0.05;0.44 | 125 | 1.56 | 0.12 |  |
|  |  | tSNR | 0.002 | -0.001;0.005 | 125 | 1.35 | 0.18 |  |
|  |  | age | -0.0015 | -0.0124;0.009 | 125 | -0.28 | 0.78 | 0.94 |
